# *Helicobacter pylori* Detection Based on Synergistic Electromagnetic and Chemical Enhancement of Surface‐Enhanced Raman Scattering in 3D Hotspot‐Activated Gold Nanorods/Nano Mica Platelets/ZnO Quantum Dots

**DOI:** 10.1002/advs.202503562

**Published:** 2025-04-23

**Authors:** Ming‐Chang Lu, Yung‐Chi Yang, Chia‐Jung Lee, Chih‐Wei Chiu

**Affiliations:** ^1^ Department of Materials Science and Engineering National Taiwan University of Science and Technology Taipei 10607 Taiwan; ^2^ Ph.D. Program in Clinical Drug Development of Herbal Medicine College of Pharmacy Taipei Medical University Taipei 11031 Taiwan

**Keywords:** gold nanorods, *Helicobacter pylori*, nano mica platelets, surface‐enhanced Raman scattering, zinc oxide quantum dots

## Abstract

Gold nanorods (AuNRs) with a controllable aspect ratio are anchored on the surface of delaminated nano mica platelets (NMPs) in the presence of a cationic interfacial activator and protective agent enabling the positive charging of the AuNR and nanohybrid surfaces. The high anionic charge and specific surface area of NMPs stabilize AuNR growth and benefit the adsorption of anionic analytes. The nanohybrids (AuNRs/NMPs) exhibit a 3D hotspot effect due to self‐assembly and feature regularly arranged AuNRs, thus enabling Raman signal enhancement and sensitive (limit of detection (LOD) = 10^−9^ m, Raman enhancement factor (EF) = 2.0 × 10^8^) and reproducible (relative standard deviation (RSD) = 8.82%) adenine detection based on surface‐enhanced Raman scattering (SERS). The further incorporation of ZnO quantum dots (QDs) affords nanohybrids (AuNRs/NMPs/ZnO QDs) that exhibit electromagnetic and chemical signal enhancement mechanisms and enable more sensitive and reproducible adenine detection (LOD = 10^−10^ m, EF = 1.6 × 10^9^, RSD = 7.66%). AuNRs/NMPs/ZnO QDs are subsequently used for the selective and sensitive SERS‐based detection of *Helicobacter pylori* (LOD = 90 CFU mL^−1^). Thus, this work paves the way for the noninvasive, nonfluorescent labeling, rapid, sensitive, selective, and reproducible detection of *H. pylori*.

## Graphical abstract

Gold nanorods hybridized with nano mica platelets and ZnO quantum dots are used for the rapid, sensitive, selective, and reproducible surface‐enhanced Raman scattering‐based detection of *Helicobacter pylori* in different scenarios. The origin of this high performance is ascribed to the synergy between the three operational enhancement mechanisms and the 3D hotspot effect due to particle self‐assembly.

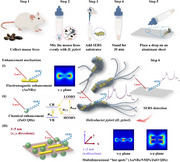

## 1 Introduction

*Helicobacter pylori* is a gram‐negative microaerobic spiral‐shaped bacterium that plays an important role in the pathogenesis of duodenal ulcerative diseases, including chronic gastritis, gastric ulcers, gastric cancer, and gastric mucosa‐associated lymphoid tissue lymphoma,^[^ ^1^, ^2^, ^3^, ^4^ ^]^ and has been extensively researched since its initial isolation from patients with gastritis by Warren and Marshall (1982).^[^ ^5^ ^]^ The *H. pylori* infection affects more than half of the global population to varying degrees and is associated with a high morbidity rate, with ≈2% of infected individuals ultimately developing gastric cancer.^[^ ^6^ ^]^ The prevalence of this infection considerably varies with the geographical region, ranging from 18.9% in Switzerland to 87.7% in Nigeria.^[^ ^7^ ^]^ At present, the diagnostic procedures for *H. pylori* infection can be classified as invasive or noninvasive, depending on whether gastric biopsy is required. Invasive procedures, including immunohistochemical staining, rapid urease tests, and bacterial culturing, primarily involve the acquisition of biopsy specimens using a gastroscope.^[^ ^8^ ^]^ Given that immunohistochemical staining is costly and time‐consuming, while culturing requires a high level of technical expertise, the widespread use of invasive methods is hindered by the need for long analysis times, expensive equipment, and operator training.^[^ ^9^ ^]^ Therefore, considerable attention has been drawn to noninvasive *H. pylori* detection methods, as exemplified by fecal antigen detection, serum antibody determination, and urea ^13^C/^14^C breath testing (UBT),^[^ ^10^ ^]^ with UBT being the most common method.^[^ ^11^ ^]^ Despite the sensitivity and relative safety of UBT, its commercialization is hindered by the introduction of the radioactive ^14^C (which cannot be excreted from the body), the associated health risks, the need for specialized equipment, and the possibility of false positive outcomes.^[^ ^12^ ^]^ Given the variable sensitivity, cost‐effectiveness, and (dis)advantages of the abovementioned methods, *H. pylori* detection should not rely on a single test,^[^ ^13^ ^]^ and developing a novel, rapid, cost‐effective, and highly sensitive detection method remains a critical challenge in the field.

Surface‐enhanced Raman scattering (SERS) is an attractive molecular identification technique providing structural information based on molecular vibration fingerprints and relies on the generation of a very strong electromagnetic field (hotspot effect) due to the localized surface plasmon resonance (LSPR) of precious‐metal nanoparticles.^[^ ^14^, ^15^, ^16^, ^17^ ^]^ Precious‐metal nanoparticles have long been used as SERS substrates because of their biocompatibility and chemical stability^[^ ^18^, ^19^ ^]^ and are widely exploited in biosensing.^[^ ^20^, ^21^ ^]^ Among the anisotropic metal nanoparticles used in biomedical research, gold nanorods (AuNRs) stand out because of their tunable aspect ratio and near‐infrared LSPR, high biocompatibility, and good stability.^[^ ^22^, ^23^, ^24^ ^]^ However, traditional colloidal AuNR‐based SERS substrates suffer from poor reproducibility and aggregation issues, significantly limiting their practical applications. Previously, we prepared fluorinated mica using one‐step delamination to form nano mica platelets (NMPs)^[^ ^25^, ^26^ ^]^ and used them as a substrate for the stable growth and stabilization of metal nanoparticles.^[^ ^27^ ^]^ The Si─O bonds on the NMP surface facilitate the adsorption of microbial molecules through polar interactions and ionic surface charges. NMPs exhibit a uniform shape and high surface‐area‐to‐volume ratio and are optimal for applications requiring strong surface interactions at the nanoscale.^[^ ^25^ ^]^ The NMP surface features a high density of anionic charges, which can be self‐assembled to generate a three‐dimensional (3D) hotspot effect and thereby enhance the Raman signal.^[^ ^28^ ^]^ Previously, this mechanism was exploited to stabilize AuNRs on the surface of nanohybrids, enhancing target analyte binding through polar adsorption^[^ ^29^, ^30^ ^]^ and enabling the development of a selective and highly sensitive SERS substrate. Furthermore, this strategic material design effectively overcomes the limitations of conventional SERS platforms by ensuring high stability, controlled nanoparticle distribution, and enhanced signal reproducibility.

Despite recent advancements, integrating semiconductor materials into SERS substrates to further improve sensitivity and biocompatibility remains largely unexplored. The construction of composite SERS substrates has drawn considerable attention and typically involves the introduction of semiconductors exhibiting high chemical stabilities, biocompatibilities, and specific surface areas. These composites are expected to be free from the drawbacks of purely precious‐metal substrates, particularly wide‐bandgap ones (e.g., ZnO and TiO_2_).^[^ ^31^, ^32^ ^]^ However, the corresponding research and development is still in its infancy, which highlights the need to gain a comprehensive understanding of SERS enhancement mechanisms. These mechanisms can be classified into the electromagnetic enhancement mechanism (EM), which is caused by LSPR, and the chemical enhancement mechanism (CM), caused by charge transfer (CT).^[^ ^33^, ^34^, ^35^, ^36^, ^37^, ^38^, ^39^ ^]^

Herein, ZnO quantum dots (QDs), which exhibit favorable biocompatibility, large specific surface area, and wide bandgap, were used to prepare AuNR‐containing nanohybrids (AuNRs/NMPs/ZnO QDs) as a substrate for *H. pylori* detection.^[^ ^40^, ^41^, ^42^, ^43^, ^44^ ^]^ The introduction of ZnO QDs not only facilitates CT‐based CM, further amplifying the SERS signal, but also enhances biocompatibility, making this nanohybrid system particularly advantageous for biomedical applications. Furthermore, this study is the first to report the application of AuNR‐based SERS detection for *H. pylori*, marking a significant breakthrough in biosensing technology. The 2D NMPs were employed to stabilize and reduce AuNRs and form nanohybrids (AuNRs/NMPs) with a 3D hotspot effect, and the SERS enhancement factor was further increased through the incorporation of ZnO QDs, which proved the importance of a dual regulatory strategy involving the EM and CM. The bacteria were captured by physisorption, which enhanced the SERS signal, and a high limit of detection (LOD, 90 CFU mL^−1^) and satisfactory sensitivity was achieved. By leveraging the synergistic effects of plasmonic enhancement, CT, and controlled nanostructure assembly, this study provides a paradigm shift in SERS‐based bacterial detection. Owing to its high sensitivity, accuracy, and rapidity, the developed method has the potential to replace traditional real‐time detection techniques (e.g., immunohistochemical staining, rapid urease tests, and UBT) and meet the requirements for *H. pylori* detection in diverse scenarios.

## 2 Results and Discussion

### 2.1 Synthesis and Analysis of AuNRs/NMPs

**Figure 1:**
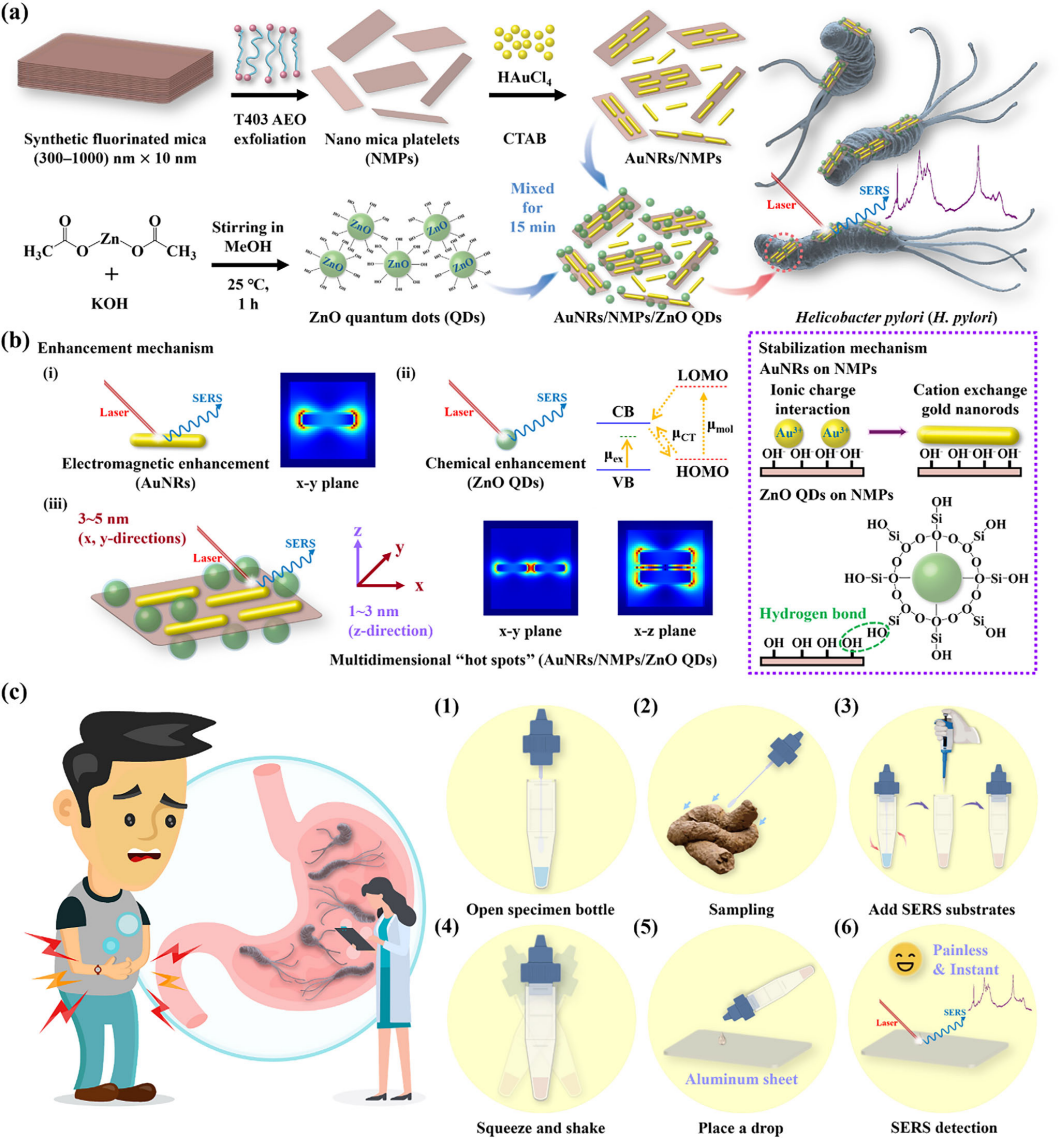
a) Schematic synthesis and b) surface‐enhanced Raman scattering (SERS) mechanisms of gold nanorods (AuNRs)/nano mica platelets (NMPs)/ZnO quantum dots (QDs). c) Improvement of the conventional fecal antigen detection method using SERS. The procedure is as follows. (1) Keep the sample bottle upright and the cap opened to avoid extract dumping. (2) Dip the sampling rod into the fecal matter at three distinct locations, making punctures at each point. (3) Thoroughly mix the sample in the bottle, add the SERS substrate and tightly fasten the bottle. (4) Squeeze and shake the bottle 20 times and leave it to stand for 30 min. (5) Place a drop of the sample/SERS substrate mixture on an aluminum plate to dry. (6) Perform the rapid and painless SERS detection of *Helicobacter pylori*.

A concise introduction is presented at the outset, summarizing the key objectives and scope of this research. **Figure** **1**a illustrates the preparation of AuNRs/NMPs/ZnO QDs. Synthetic fluorinated mica was subjected to cation‐exchange and polymerization reactions using a delaminating agent (T403AEO) to facilitate the formation of monolithic NMPs, and the polymer residues on their surface were subsequently removed by extraction and filtration. The charged surface of the NMPs facilitated a cation‐exchange reaction with gold particles, leading to the electrostatic attraction between the negatively charged NMP surface and the cetyltrimethylammonium bromide (CTAB)‐stabilized Au seeds, thereby stabilizing the reductive formation of AuNRs on the NMP surface through a seed‐mediated method. The resulting nanohybrids (AuNRs/NMPs) were decorated with ZnO QDs to form AuNRs/NMPs/ZnO QDs. The SERS enhancement effect of AuNRs/NMPs/ZnO QDs responsible for the detection of *H. pylori* was due to the i) EM due to the LSPR of AuNRs, ii) CM due to the CT effect of ZnO QDs, and iii) promotional effect of NMPs on the stable reduction of AuNRs and their dispersion and alignment (caused by the large ionic charge on the NMP surface) (Figure 1b). Further Raman signal intensification was achieved through the 3D hotspot effect due to particle self‐assembly. The *H. pylori* infection is a pervasive global health concern and is strongly correlated with several gastrointestinal disorders and complications (including indigestion and the associated abdominal discomfort, flatulence, belching, and nausea) while playing a pivotal role in the pathogenesis of gastric cancer. Current *H. pylori* testing methods differ in terms of sensitivity, cost‐effectiveness, and other (dis)advantages. In our future work, the results of this study will be employed to improve conventional fecal antigen detection methods using SERS (Figure 1c). Specifically, we will enhance detection sensitivity and specificity while reducing the likelihood of false positives. By leveraging the characteristic peak assessment of SERS spectra, rapid, painless, highly sensitive, and reproducible *H. pylori* detection will be realized to markedly enhance diagnostic precision and patient comfort.

AuNRs with a uniform size distribution were synthesized via a modified seed‐mediated method (Figure S1a, Supporting Information),^[^ ^45^ ^]^ wherein HAuCl_4_ was reduced by NaBH_4_ in the presence of CTAB to generate gold seeds. Subsequent growth in a CTAB‐stabilized solution was optimized by introducing Ag^+^, which modulated the (100)/(110) growth rate ratio and controlled the aspect ratio through underpotential deposition.^[^ ^46^ ^]^ Preliminary identification was conducted using a UV–vis spectrometer, with absorption spectra collected at 1 min intervals throughout the reduction process. The stabilized generation of AuNRs was achieved within ≈120 min, which markedly shortened the overall reduction process (Figure S1b, Supporting Information). The role of Ag^+^ as an essential intermediate is of paramount importance. The aspect ratio of the AuNRs could be effectively adjusted by varying the amount of added AgNO_3_ (0.00–0.10 mL). The maximum absorption wavelength of AuNRs increased with their increasing aspect ratio (Figure S1c, Supporting Information), which indicated that the position of surface plasmon resonance (SPR) peaks could be adjusted by controlling the size of the AuNRs through Ag^+^ concentration control. Figure S1d (Supporting Information) presents the linear fit of a plot of the mean aspect ratio versus absorption peak position (*R* ^2^ = 0.9922), revealing a strong linear correlation between these two parameters. Figure S1e (1)–(5) (Supporting Information) presents the transmission electron microscopy (TEM) images of AuNRs with different aspect ratios. Figure S1e (6)–(10) (Supporting Information) presents the corresponding distributions and averages determined using the Image J software, revealing that the AuNRs had a narrow size distribution and uniform size. AuNRs with average aspect ratios of 1.84–4.65 could be obtained at various Ag^+^ concentrations, and the related UV–vis absorption maxima and average aspect ratios are presented in Table S1 (Supporting Information). The SERS enhancement effect is contingent upon the LSPR of precious metals, which is affected by the shape and size of the corresponding nanoparticles and the distance between them. Accordingly, the impact of nanoparticle dimensions on LSPR was assessed by modifying the aspect ratios of AuNRs. Herein, SERS analysis was conducted to detect adenine as a representative biomolecule. Figure S1f (Supporting Information) shows the SERS signals detected at an excitation wavelength of 785 nm for AuNRs with varying aspect ratios at a constant concentration (10^−4^ m) of adenine. The optimal SERS signal (signal‐to‐background (S/B) ratio = 2.0) was obtained at an average aspect ratio of 4.12. Figure S2 (Supporting Information) presents the effects of the AuNR aspect ratio on the integrated intensity of characteristic peaks within the 704–764 cm^−1^ range, revealing that this intensity was highest (1.32 × 10^5^) at an average aspect ratio of 4.12, with the individual SERS intensity and S/B ratios presented in Table S1 (Supporting Information). The primary reason for this behavior is that the SERS signal was maximally enhanced when the aspect ratio‐dependent maximum absorption wavelength of the SPR on the longitudinal axis of the AuNRs overlapped with the wavelength of the excitation light source (785 nm laser). This observation is consistent with our finite‐difference time‐domain (FDTD) simulations, which predict a similar enhancement effect (Figure S3, Supporting Information). Accordingly, AuNRs with an average aspect ratio of 4.12 were chosen for subsequent experiments, with the corresponding adenine LOD determined as 10^−7^ m, indicating high sensitivity (Figure S1g, Supporting Information). The linear fit of the log(integrated intensity in the range of 704–764 cm^−1^)–log(adenine concentration) plot featured an *R* ^2^ value of 0.9787 (Figure S1h, Supporting Information).

**Figure 2:**
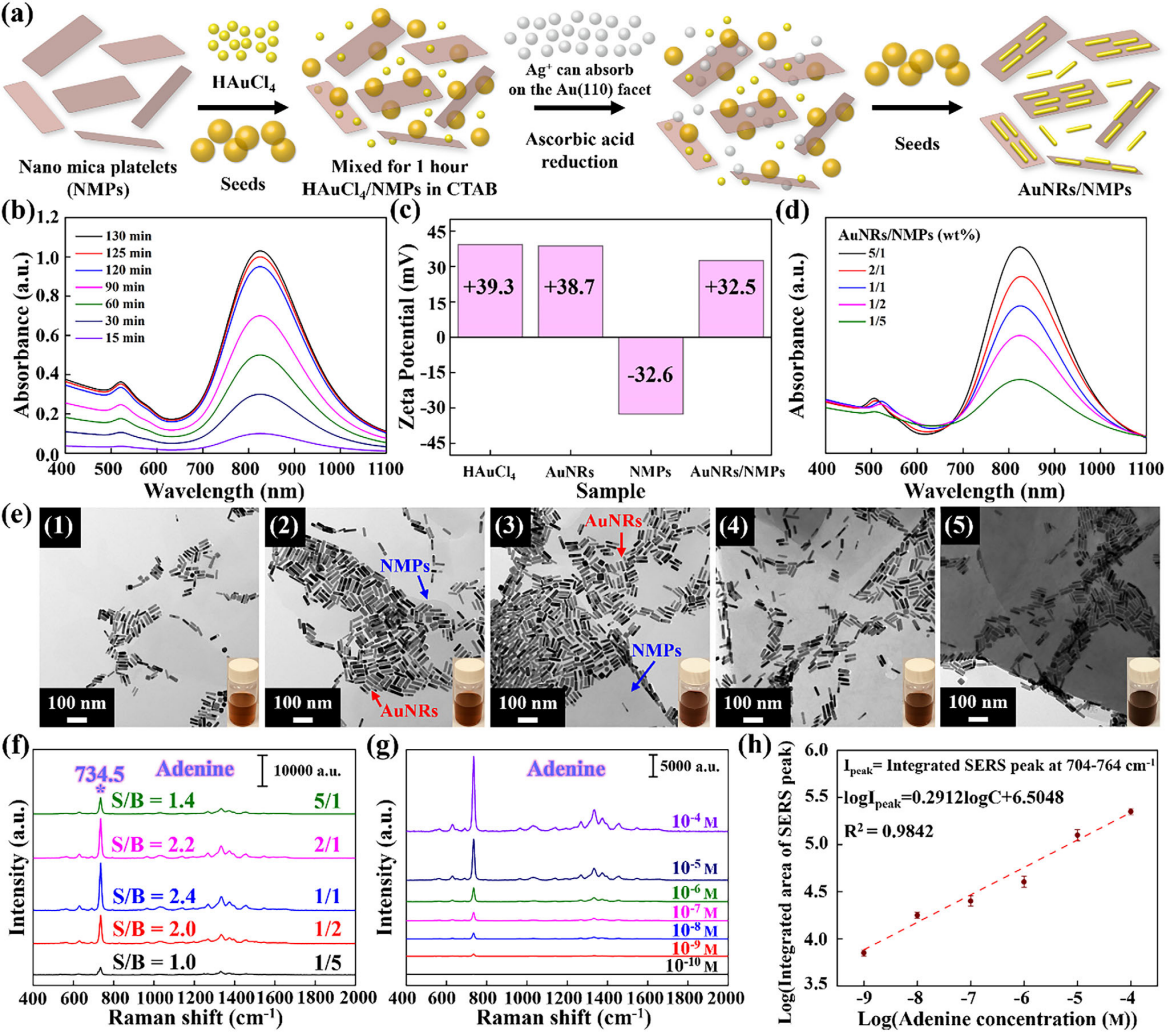
a) Generation of AuNRs on the surface of NMPs using a seed‐mediated method. b) UV–vis spectra of AuNRs/NMPs (1/1, w/w) recorded at different reduction times (*n* = 10). The spectrum corresponding to the median set was selected for representative plotting. c) Zeta potentials of HAuCl_4_, AuNRs, NMPs, and AuNRs/NMPs (1/1, w/w). d) UV–vis spectra of AuNRs/NMPs with different component weight ratios (*n* = 10). e) Transmission electron microscopy images of AuNRs/NMPs with weight ratios of (1) 5/1, (2) 2/1, (3) 1/1, (4) 1/2, and (5) 1/5 (insets show photographs of corresponding dispersions). f) SERS responses of AuNRs/NMPs with different weight ratios to adenine (10^−4^ m). Spectra were averaged over 50 randomly selected positions (*n* = 50), with the median set displayed. g) SERS responses of AuNRs/NMPs (1/1, w/w) to different adenine concentrations (*n* = 50). h) Linear correlation between log(integrated intensity in the range of 704–764 cm^−1^) and log(adenine concentration).

Layered silicate clay is a 2D layered nanomaterial with exceptional mechanical and self‐alignment properties, which can be attributed to its structure and cation‐exchange characteristics.^[^ ^47^ ^]^ The exchange of cations (e.g., Na^+^, K^+^, and Ca^2+^) between clay layers can effectively stabilize nanoparticles.^[^ ^48^, ^49^ ^]^ Herein, we used a synthetic fluorinated mica with a high aspect ratio, exhibiting lateral dimensions of 300–1000 nm and a thickness of 1 nm, along with exemplary mechanical properties and self‐alignment capability. **Figure** **2**a depicts a schematic of NMPs acting as a growth carrier for stabilized AuNRs. The charged surface of the NMPs facilitated the cation‐exchange reaction of gold particles, leading to the reduction of AuNRs on the NMP surface and the formation of AuNRs/NMPs. Figure 2b illustrates the UV–vis absorption spectra recorded at different reduction times, revealing that reduction was complete after ≈125 min. The proposed method (Video S1, Supporting Information) is considerably faster and cheaper than conventional techniques of AuNR nanohybrid production. The surface potentials of AuNRs/NMPs and their precursors were determined using zeta potential analysis (Figure 2c). The variation in surface potential values indirectly confirmed the stable reduction of AuNRs on the NMP surface. Figure 2d shows the UV–vis spectra of AuNRs/NMPs produced at AuNR/NMP weight ratios of 5/1, 2/1, 1/1, 1/2, and 1/5. When the NMP content was insufficient, the particles tended to agglomerate and adopt irregular shapes, which reduced the yield of AuNRs. As the NMP content increased, the growth of AuNRs was influenced by the accumulation of excessive ionic charge. The absorption peak weakened and broadened with the increasing NMP content, indicating that the AuNR size and shape concomitantly became more heterogeneous. TEM imaging (Figure 2e) indicated that the reduction stability of the anisotropic AuNRs was compromised when the NMPs content was excessive or insufficient, with the best dispersion and alignment achieved at a weight ratio of 1/1 (Figure 2e(3)). Energy‐dispersive X‐ray spectroscopy (EDS) analysis (Figure S4, Supporting Information) showed that the surface of AuNRs/NMPs mainly contained Au, while the NMPs contained Si, O, and Mg. The adenine detection efficiency of AuNRs/NMPs with different component ratios was evaluated at an adenine concentration of 10^−4^ m (Figure 2f) and was optimal (S/B ratio = 2.4, Table S2, Supporting Information) at a weight ratio of 1/1. The integrated intensity of adenine peaks within the 704–764 cm^−1^ range (Figure S5 and Table S2, Supporting Information) was also maximal for the 1/1 ratio (2.24 × 10^5^), which demonstrated that the SERS signal intensity was strongly influenced by the number, arrangement, and spacing of precious‐metal nanoparticles. A key feature of the NMPs is their ultrathin nature, which enables AuNRs to be adsorbed on both surfaces. This structural advantage facilitates a self‐assembly process that enhances interparticle coupling and promotes the formation of hierarchical nanostructures with controlled interparticle gaps extending along the z‐axis. The resulting 3D hotspot effect significantly amplifies localized electromagnetic fields, thereby enhancing the Raman signal, as demonstrated by the adenine detection results (Figure S6, Supporting Information). Furthermore, FDTD simulations confirm that AuNRs/NMPs exhibit superior field localization and stronger signal amplification compared to AuNRs/Mica (Figure S7, Supporting Information), highlighting the critical role of NMPs in optimizing the plasmonic architecture for highly sensitive and reproducible SERS‐based detection. Finally, the substrate with the optimal detection efficacy (aspect ratio = 4.12, weight ratio = 1/1) was used for adenine detection, and the corresponding LOD was determined as 10^−9^ m (Figure 2g). The linear fit of the log(integrated intensity in the range of 704–764 cm^−1^)–log(adenine concentration) plot featured *R* ^2^ = 0.9842 (Figure 2h). Sensitivity was quantified in terms of the surface enhancement factor (EF), which is defined as follows (Equation 1)^[^ ^50^, ^51^ ^]^:

(1) $$\mathrm{EF}=\left(I_{\mathrm{SERS}}/C_{\mathrm{SERS}}\right)/\left(I_{\mathrm{norm}}/C_{\mathrm{norm}}\right)$$

where *C*_norm_ is the original adenine concentration in the laser focal length range (0.1 m), *C*_SERS_ is the adenine LOD of the SERS substrate, *I*_norm_ is the general Raman intensity, and *I*_SERS_ is the integrated SERS signal intensity in the range of 704–764 cm^−1^. Under optimal conditions, the EF reached 2.0 × 10^8^. The aforementioned results demonstrate that AuNRs produced in the presence of CTAB as a protective agent and deposited on NMPs exhibited a relatively regular arrangement, which minimized agglomeration and helped control the distance between the particles during reduction (Figure 2e (3)). Furthermore, the strong localized electromagnetic field effect at the endpoints of AuNRs could markedly enhance SERS sensitivity.^[^ ^52^ ^]^

### 2.2 Preparation and SERS Detection of AuNRs/NMPs/ZnO QDs

**Figure 3:**
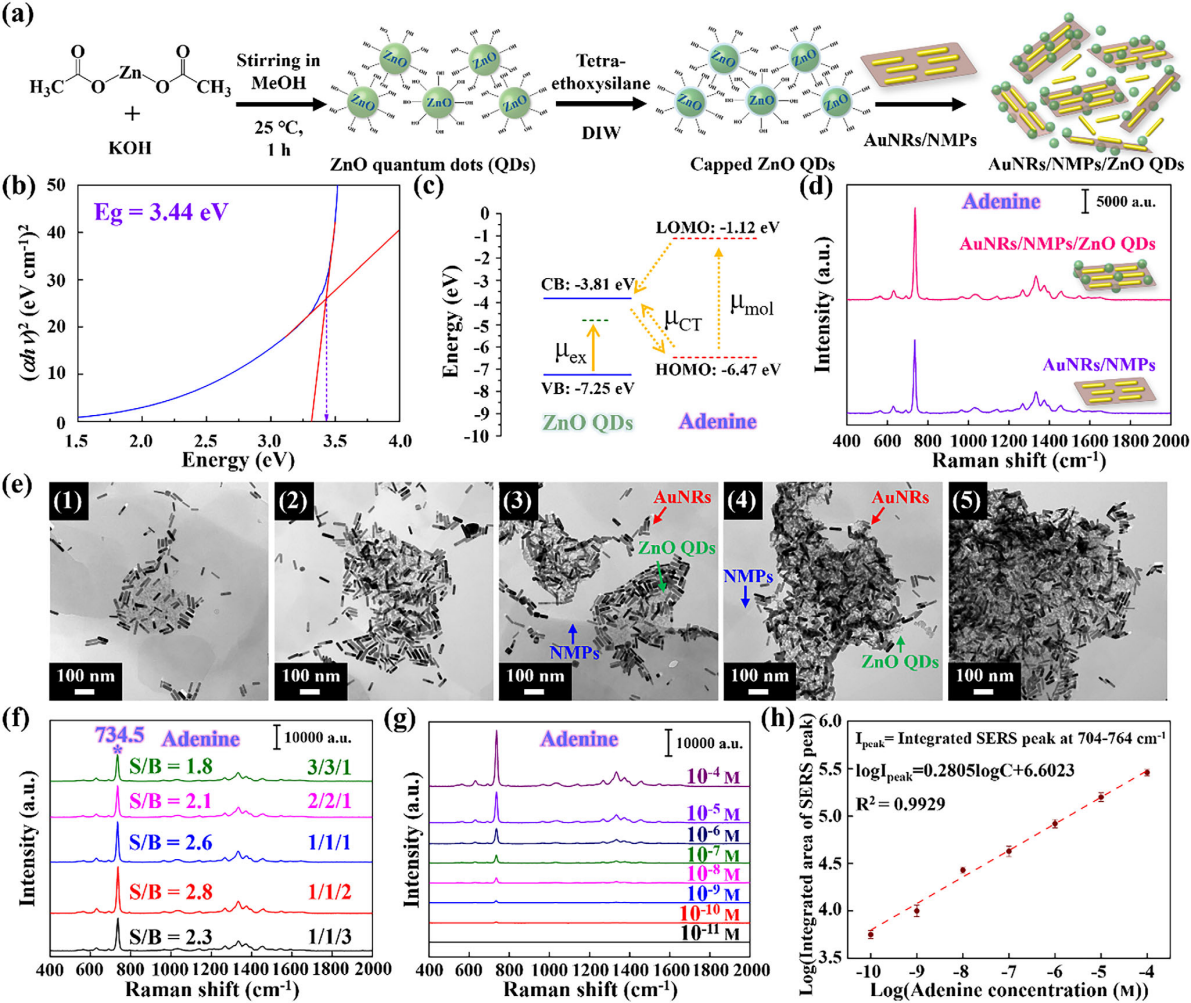
a) Schematic synthesis of AuNRs/NMPs/ZnO QDs. b) Tauc plot of ZnO QDs. c) Energy level diagram illustrating charge transfer between ZnO QDs and adenine. d) SERS responses of AuNRs/NMPs/ZnO QDs (1/1/2, w/w) and AuNRs/NMPs (1/1, w/w) to adenine (10^−4^ m). Spectra were averaged over 50 randomly selected positions (*n* = 50), with the median set displayed. e) Transmission electron microscopy images illustrating the distribution patterns of AuNRs, NMPs, and ZnO QDs in AuNRs/NMPs/ZnO QDs with weight ratios of (1) 3/3/1, (2) 2/2/1, (3) 1/1/1, (4) 1/1/2, and (5) 1/1/5. f) SERS responses of AuNRs/NMPs/ZnO QDs with different weight ratios to adenine (10^−4^ m; *n* = 50). g) SERS responses of AuNRs/NMPs/ZnO QDs (1/1/2, w/w) to different adenine concentrations (*n* = 50). h) Linear fit of the log(integrated intensity in the range of 704–764 cm^−1^)–log(adenine concentration) plot.

SERS relies on EM and CM, with the latter being more important for semiconductor substrates^[^ ^53^ ^]^ and primarily relying on CT and exciton resonance enhancement. When the lowest unoccupied molecular orbital (LUMO) and highest occupied molecular orbital (HOMO) energies of the analyte align with the conduction band (CB) and valence band (VB) energies of the substrate, effective CT occurs, changing the polarization and electron density distribution of the molecule and thus resulting in SERS.^[^ ^54^ ^]^ Therefore, SERS substrates capable of amplifying the weak Raman signals of trace analytes through the combined action of both mechanisms are highly sought after. The semiconductor employed herein (ZnO) exhibited a wide bandgap and favorable biocompatibility,^[^ ^55^, ^56^, ^57^ ^]^ which enabled the CT‐based CM and efficient SERS‐based biomolecule detection. **Figure** **3**a illustrates the preparation of AuNRs/NMPs/ZnO QDs. The hydroxyl groups on the NMP surface were rich in silanol (Si─OH) groups, which were generated during delamination through the hydrolysis of surface siloxyl groups and enabled the binding and adsorption of ZnO QDs through strong hydrogen bonding. The CM of ZnO QDs was probed by UV–vis spectrometry (Figure S8, Supporting Information), with the absorption edge at 360 nm used to determine the capacity to facilitate electron transfer. Based on the corresponding Tauc plot, the bandgap of ZnO QDs was determined as 3.44 eV (Figure 3b). To investigate the CT between ZnO QDs and surface‐adsorbed adenine, we examined the corresponding energy levels (Figure 3c). The CB and VB energies of ZnO QDs were located at ≈−3.81 and −7.25 eV, respectively,^[^ ^58^ ^]^ while the LUMO and HOMO energies of adenine were located at −1.12 and −6.47 eV, respectively.^[^ ^59^ ^]^ The Raman enhancement observed in the ZnO QD–adenine system was attributed to the molecular resonance of adenine (*µm*_ol_), photon‐induced CT resonance, and ground‐state CT resonance between the energy levels of adenine and ZnO QDs (*µ*_CT_), and the excitation resonance of the surface‐state energy level (*µ*_ex_) (Figure 3c). Upon excitation, the electrons in the adenine HOMO were transferred to the corresponding LUMO and, subsequently, to the CB of ZnO QDs. Similarly, the excitation of electrons in the HOMO of ZnO led to their transfer to the CB of the QDs. Additionally, electrons in the VB of ZnO QDs were excited to the surface state to result in CT and Raman signal enhancement. The role of ZnO QDs in CM was further confirmed by control experiments, as shown in Figure S9 (Supporting Information), where the LOD for adenine using ZnO QDs alone was determined to be 10^−5^ m, with a corresponding EF of 7.5 × 10^4^. These results provide direct evidence that ZnO QDs can effectively enhance the Raman signal through the CM mechanism by promoting CT interactions between the analyte and the semiconductor surface. Figure 3d presents the SERS responses of AuNRs/NMPs/ZnO QDs (1/1/2, w/w/w) and AuNRs/NMPs (1/1, w/w) to adenine (10^−4^ m), showing that the CM resulting from the CT of ZnO QDs enhanced detection sensitivity. Figure S10 (Supporting Information) depicts the SERS responses of AuNRs/NMPs/ZnO QDs (1/1/2, w/w/w) and AuNRs/NMPs (1/1, w/w) to three additional DNA bases, namely thymine, guanine, and cytosine. The corresponding spectra featured characteristic peaks at 795/1601, 661, and 790 cm^−1^, respectively, and ZnO QD incorporation resulted in a higher detection sensitivity in all cases. Thus, AuNRs/NMPs/ZnO QDs enabled sensitive and selective biomolecule detection and, potentially, *H. pylori* detection. To investigate the interactions between AuNRs/NMPs/ZnO QDs in terms of the EM and CM, we examined samples with AuNR/NMP/QD weight ratios of 3/3/1, 2/2/1, 1/1/1, 1/1/2, and 1/1/3. TEM imaging revealed that overly high and low ZnO contents resulted in insufficient adsorption around AuNRs/NMPs, thereby hindering SERS signal enhancement (Figure 3e). At the optimal weight ratio of 1/1/2 (Figure 3e (4)), effective QD dispersion and alignment enabled efficient adsorption around AuNRs/NMPs. The elemental composition of AuNRs/NMPs/ZnO QDs was probed by EDS (Figure S11, Supporting Information). NMPs were found to contain Si, O, Mg, and other elements, while ZnO QDs were found to contain Zn and O. Figure 3f illustrates the SERS responses of AuNRs/NMPs/ZnO QDs with different compositions to adenine (10^−4^ m), showing that the optimal signal intensity (signal‐to‐noise ratio = 2.8) was obtained at a weight ratio of 1/1/2, with the integrated intensity of the adenine peaks in the 704–764 cm^−1^ range under these conditions reaching 2.88 × 10^5^ (Figure S12 and Table S3, Supporting Information). The adenine LOD of the optimal substrate was determined as 10^−10^ m (Figure 3g). The linear fit of the log(integrated intensity in the range of 704–764 cm^−1^)–log(adenine concentration) plot featured *R* ^2^ = 0.9929 (Figure 3h), and EF corresponding to above mentioned LOD was determined as 1.6 × 10^9^. The superior Raman signal enhancement observed for AuNRs/NMPs/ZnO QDs can be attributed to the synergistic interplay between EM and CM mechanisms. The LSPR of AuNRs plays a crucial role in generating a highly concentrated electromagnetic field, significantly amplifying the Raman scattering of nearby analytes. Meanwhile, ZnO QDs introduce an additional enhancement pathway through CT, facilitating electron migration between the CB of ZnO and the molecular orbitals of the analyte, thereby increasing molecular polarizability and further boosting Raman signal intensity. Moreover, the inclusion of NMPs contributes to a well‐defined 3D hotspot effect by enabling the controlled self‐assembly and dispersion of AuNRs, which optimizes the interparticle spacing and maximizes localized EM field intensity. Collectively, these synergistic effects lead to an exceptionally high SERS EF and detection sensitivity. To further substantiate the individual contributions of these mechanisms, Table S4 (Supporting Information) presents a comparative analysis of SERS‐based adenine detection among different material compositions, illustrating the significant role of both LSPR‐induced EM and CT‐induced CM in achieving superior detection performance. This comprehensive understanding of the SERS enhancement mechanism validates the rational design of our hybrid nanostructure and highlights its potential for rapid, ultrasensitive, and reproducible biomolecular detection (Video S2, Supporting Information). Additionally, we examined the uniformity and stability of the SERS intensity distribution for AuNRs, AuNRs/NMPs, and AuNRs/NMPs/ZnO QDs. Figure S13a (Supporting Information) depicts the signal intensities (10^−4^ m adenine, 734.5 cm^−1^) for 50 points randomly selected on AuNRs and shows that the corresponding relative standard deviation (RSD) equaled 12.8%, while Figure S13b (Supporting Information) illustrates the corresponding intensity distribution (SERS mapping) over a 400 µm^2^ area. Figure S13c (Supporting Information) depicts the signal intensities (10^−4^ m adenine, 734.5 cm^−1^) for 50 points randomly selected on AuNRs/NMPs and shows that the corresponding relative standard deviation (RSD) equaled 8.82%, while Figure S13d (Supporting Information) illustrates the corresponding intensity distribution (SERS mapping) over a 400 µm^2^ area. Figure S13e (Supporting Information) depicts the signal intensities (10^−4^ m adenine, 734.5 cm^−1^) for 50 points randomly selected on AuNRs/NMPs and shows that the corresponding relative standard deviation (RSD) equaled 7.66%, while Figure S13f (Supporting Information) illustrates the corresponding intensity distribution (SERS mapping) over a 400 µm^2^ area. The RSD markedly decreased upon going from AuNRs to AuNRs/NMPs, which indicated that NMPs facilitated the uniform dispersion and stabilization of AuNRs and thereby markedly enhanced detection stability. The concomitant increase in the Raman signal intensity over the examined area was attributed to the 3D hotspot effect caused by the self‐assembly of NMPs. The incorporation of ZnO QDs further decreased the RSD and increased the signal intensity. The primary mechanism responsible for this behavior was identified as CM resulting from CT. Furthermore, ZnO QDs were stably adsorbed on the NMP surface through hydrogen bonding, which also contributed to the more uniform signal intensity distribution. This finding suggested that AuNRs/NMPs/ZnO QDs exhibited excellent reproducibility and stability.

### 2.3 Interaction of AuNRs/NMPs/ZnO QDs with *H. pylori* and SERS Detection

**Figure 4:**
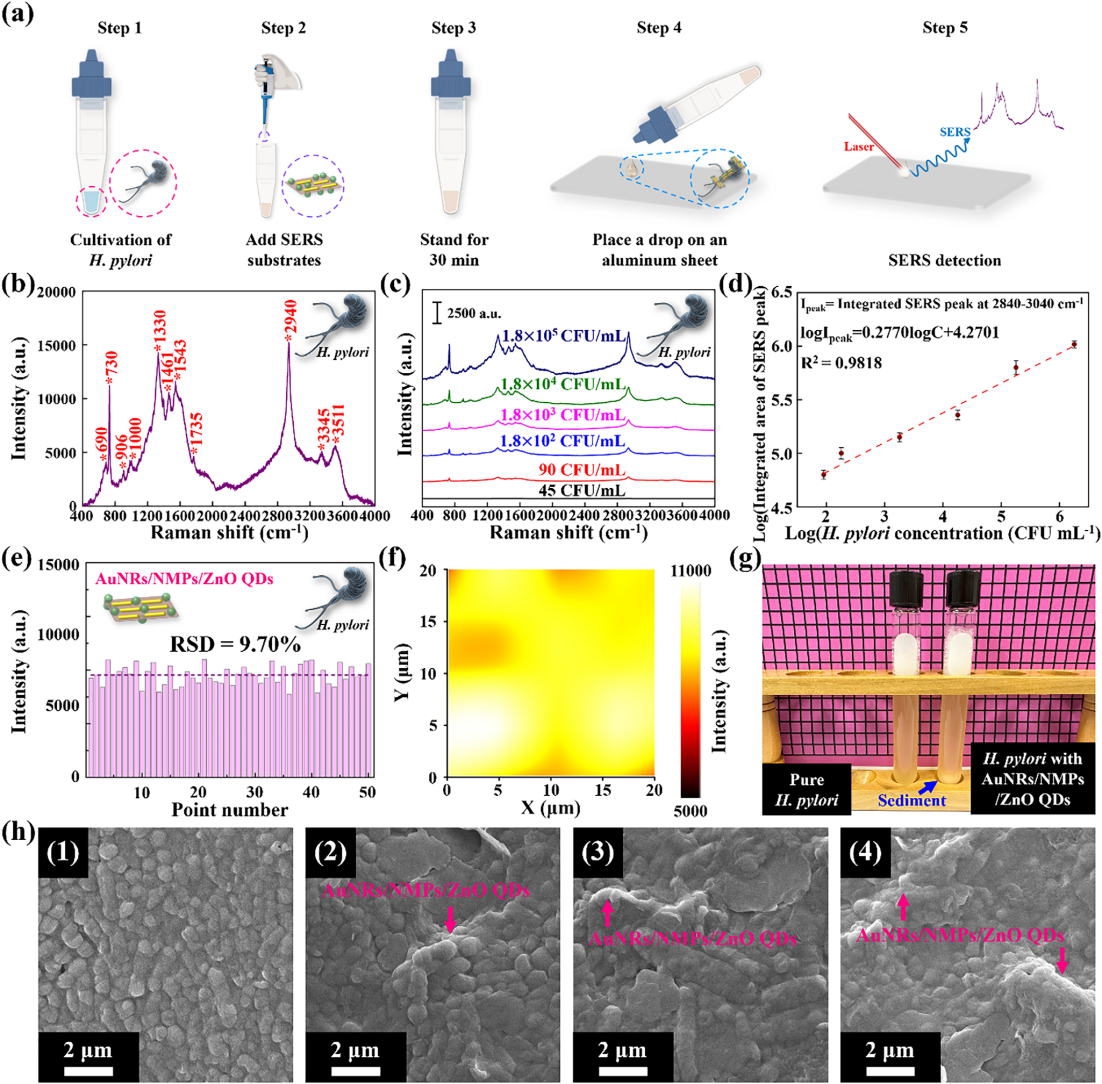
a) Flow chart of the SERS‐based detection of *H. pylori* by AuNRs/NMPs/ZnO QDs. b) SERS response of AuNRs/NMPs/ZnO QDs (1/1/2, w/w) to *H. pylori* (1.8 × 10^7^ CFU mL^−1^). c) SERS responses of AuNRs/NMPs/ZnO QDs (1/1/2, w/w) to different *H. pylori* concentrations. Spectra were averaged over 50 randomly selected positions (*n* = 50), with the median set displayed. d) Linear fit of the log(integrated intensity in the range of 2840–3040 cm^−1^)– log(*H. pylori* concentration) plot. e) Raman signal intensities of *H. pylori* (1.8 × 10^6^ CFU mL^−1^, 2940 cm^−1^) at 50 randomly selected points on AuNRs/NMPs/ZnO QDs. f) Distribution of the Raman signal intensity of *H. pylori* (1.8 × 10^6^ CFU mL^−1^, 2940 cm^−1^) over a 400 µm^2^ area of AuNRs/NMPs/ZnO QDs. g) Physical capture of bacteria by AuNRs/NMPs/ZnO QDs and resulting precipitation. h) Scanning electron microscopy image of the precipitate in (g). The white wrinkled portion represents the SERS substrate. Image (1) refers to *H. pylori* without any substrate after seven days of culturing, while other images refer to precipitates formed after standing for (2) 10, (3) 20, and (4) 30 min.

**Figure** **4**a depicts the flow chart of the SERS‐based detection of *H. pylori* by AuNRs/NMPs/ZnO QDs. Figure 4b presents the SERS spectrum corresponding to the detection of *H. pylori* (1.8 × 10^7^ CFU mL^−1^) by AuNRs/NMPs/ZnO QDs (1/1/2, w/w), with the related peak assignments provided in Table S5 (Supporting Information). The corresponding LOD was determined as 90 CFU mL^−1^ (Figure 4c), and the linear fit of the log(intensity)–log(concentration) plot featured *R* ^2^ = 0.9818 (Figure 4d). Thus, the developed method was concluded to have the advantages of a high sensitivity, large linearity range, and fast detection. The enhanced signal was quantified at 50 random points, and the corresponding RSD (9.70%) indicated excellent reproducibility (Figure 4e). The distribution of the Raman signal intensity for *H. pylori* (1.8 × 10^6^ CFU mL^−1^) at 2940 cm^−1^ over a 400 µm^2^ area of AuNRs/NMPs/ZnO QDs was fairly uniform (Figure 4f). *H. pylori* is a gram‐negative bacterium with an outer membrane featuring abundant negatively charged head groups, such as those of lipopolysaccharides and phosphoglycerol, on the outermost leaflet of the lipid bilayer.^[^ ^60^ ^]^ Given that AuNRs/NMPs/ZnO QDs were synthesized using CTAB as a protective agent, they exhibited a positively charged surface well suited for the adsorption of negatively charged molecules. Figure 4g illustrates the physical capture of *H. pylori* by the substrate. Following a seven‐day culturing period, the bacteria were washed, centrifuged in phosphate‐buffered saline, dehydrated in ethanol, and introduced to the substrate, and the mixture was allowed to stand for a designated time. The formation of a precipitate confirmed the binding of *H. pylori* (Video S3, Supporting Information), and the precipitate was characterized by field‐emission scanning electron microscopy. As illustrated in Figure 4h, the SERS substrate exhibited clear adherence to *H. pylori* (white protrusions). Figure 4h (1) depicts the original *H. pylori* without any substrate cultured for seven days. As the contact time increased, so did the amount of the SERS substrate surrounding *H. pylori*. This observation corroborates the hypothesis that *H. pylori* can be readily captured by physical adsorption for optimal detection. Figure S14a (Supporting Information) presents the SERS responses of adenine (10^−4^ m) detected by AuNRs/NMPs/ZnO QDs after different storage durations. Even after 90 days of storage, the Raman signal intensity at 734.5 cm^−1^ retained 77% of its original value (Figure S14b, Supporting Information), confirming the long‐term stability of AuNRs/NMPs/ZnO QDs. This enhanced stability overcomes the limitations of conventional *H. pylori* detection methods, which often suffer from reagent degradation and signal attenuation over time, thereby improving the feasibility of long‐term diagnostic applications. To further assess the bacterial detection sensitivity of AuNRs/NMPs/ZnO QDs, we extended the analysis to *Staphylococcus aureus* and *Escherichia coli*. The LOD for *S. aureus* was determined to be 10 CFU mL^−1^ (Figure S15a, Supporting Information), with a corresponding linear fit (*R* ^2^ = 0.9791) in the log(intensity)–log(concentration) plot (Figure S15b, Supporting Information). Similarly, the LOD for *E. coli* was found to be 50 CFU mL^−1^ (Figure S16a, Supporting Information), with a linear correlation of *R* ^2^ = 0.9801 (Figure S16b, Supporting Information), demonstrating the high sensitivity and robust detection capability of AuNRs/NMPs/ZnO QDs across different bacterial species. Figure S17 (Supporting Information) illustrates the detection of *H. pylori* (1.8 × 10^4^ CFU mL^−1^) in the presence of *E. coli* using AuNRs/NMPs/ZnO QDs (1/1/2, w/w). Compared to the pure *H. pylori* sample, the Raman spectrum of the bacterial mixture exhibited an increased peak intensity at 730 cm^−1^, which can be attributed to the overlapping characteristic peak of *E. coli* at the same wavenumber. This result highlights the ability of the SERS probe to selectively detect *H. pylori* even in complex bacterial environments. To further enhance species differentiation, principal component analysis (PCA) was applied (Figure S18, Supporting Information). The loading distributions along LD1 and LD2 effectively distinguished *S. aureus* and *E. coli*, demonstrating that spectral variations can be utilized for bacterial classification. Notably, *H. pylori* exhibited distinct spectral features that enabled direct identification without the need for additional statistical processing. However, in cases where spectral similarities are present, PCA‐based classification offers an additional layer of precision in distinguishing bacterial species. To gain further insights into the physisorption capacity of AuNRs/NMPs/ZnO QDs and the specificity and reproducibility of *H. pylori* detection, we performed tests using a mixture of mouse feces and *H. pylori* (Figure S19a, Supporting Information). The animal experimental protocols received approval from the Institutional Animal Care and Use Committee of Taipei Medical University (LAC‐2020‐0265). The physisorption mechanism enabled the AuNRs/NMPs/ZnO QDs to capture *H. pylori* in complex matrices, facilitating rapid and accurate detection. This illustrates the prospective utility of this substrate for future SERS applications to human samples, which is anticipated to enhance the sensitivity and specificity of conventional fecal antigen detection methods. Figure S19b (Supporting Information) illustrates the results of *H. pylori* (1.8 × 10^4^ CFU mL^−1^) analysis in mouse feces performed using AuNRs/NMPs/ZnO QDs (1/1/2, w/w). The ability of this probe to detect *H. pylori* even in complex matrices indicated an excellent detection performance. The LOD of *H. pylori* in mouse fecal solutions was determined at 90 CFU mL^−1^ (Figure S19c, Supporting Information), indicating the high sensitivity of the developed method in challenging environments. Additionally, we examined the reproducibility of this method (Figure S19d, Supporting Information). Ten mice were selected, and their fecal samples were thoroughly mixed with *H. pylori* at a concentration of 1.8 × 10^4^ CFU mL^−1^. The SERS signals were randomly measured at five points of each sample, and the corresponding RSD (13.8%) indicated high stability and reliability in practical applications. Furthermore, we compared the performance of AuNRs/NMPs/ZnO QDs with that of AuNRs for the SERS‐based detection of *H. pylori* in mouse fecal solutions (Figure S20, Supporting Information). The former substrate exhibited markedly superior performance in terms of SERS signal intensity and characteristic peak specificity, which was attributed to the EM due to AuNRs, CM due to ZnO QDs, the 3D hotspot effect caused by NMPs, and the physisorption of bacteria on the substrate. The synergy between these mechanisms markedly amplified the intensity of the SERS signal, thereby enhancing detection sensitivity and accuracy. Finally, we collated and compared recent reports on the detection of adenine and bacteria using gold nanoparticle hybrids (Table S6, Supporting Information), revealing the superiority of our method in terms of the LOD and EF. Current methods for detecting *H. pylori* exhibit significant variability in sensitivity, cost‐effectiveness, and practicality, each presenting inherent limitations. The UBT, which relies on urease activity, may yield false negatives in patients undergoing proton pump inhibitor or antibiotic treatments.^[^ ^61^ ^]^ Similarly, fecal antigen detection is often compromised by antigen degradation and batch‐to‐batch variability, diminishing its reliability over time.^[^ ^62^ ^]^ While invasive biopsy‐based techniques offer high specificity, they necessitate endoscopic procedures, rendering them impractical for routine screening.^[^ ^63^ ^]^ In contrast, the proposed AuNRs/NMPs/ZnO QDs platform represents a paradigm shift in *H. pylori* detection by providing a rapid, non‐invasive, and highly sensitive alternative. By leveraging a synergistic combination of bacterial physisorption, localized plasmonic enhancement, and SERS‐based molecular fingerprinting, this approach circumvents the need for enzymatic or immunological dependencies, thereby minimizing false positives and ensuring superior detection accuracy. Furthermore, the scalable and cost‐effective synthesis of this platform enhances its translational potential, making it a promising candidate for clinical diagnostics and point‐of‐care applications.

## 3 Conclusion

AuNRs/NMPs/ZnO QDs were used for the SERS detection of *H. pylori*, exhibiting multiple signal enhancement mechanisms, including the i) EM due to the LSPR of AuNRs, ii) CM due to the CT of ZnO QDs, and iii) NMPs stabilizing the reduction of AuNRs with good dispersion and alignment because of the large ionic charge of their surface. The 3D hotspot effect due to particle self‐assembly resulted in further Raman signal enhancement. AuNRs were prepared by a seed‐mediated method and anchored on the surface of 2D delaminated NMPs. The average aspect ratio of these AuNRs was controlled in the range of 1.84–4.65 by changing the concentration of Ag^+^. Furthermore, NMPs featured a large ionic charge and specific surface area, which facilitated the stabilization of AuNR growth. The NMP‐bound AuNRs (AuNRs/NMPs) exhibited a 3D hotspot effect due to self‐assembly, thereby enhancing the Raman signal. Additionally, NMPs provided a platform for the stable adsorption of ZnO QDs and fabrication of AuNRs/NMPs/ZnO QDs. These hybrids exhibited EM due to LSPR and CM due to CT, enabling the highly sensitive SERS‐based detection of adenine (LOD = 10^−10^ m, EF = 1.6 × 10^9^, RSD = 7.66%) and *H. pylori* (LOD = 90 CFU mL^−1^). Notably, *H. pylori* capture and detection was possible even in the case of complex matrices, which indicated the high potential of AuNRs/NMPs/ZnO QDs for the rapid, sensitive, selective, and reproducible detection of *H. pylori* in different scenarios.

## 4 Experimental Section

The experimental and characterization details have been provided in the Supporting Information.

### Statistical Analysis

UV–vis absorbance spectra were recorded from ten independent replicates (*n* = 10), and the results were presented as mean values with standard deviation (SD). All quantitative SERS measurements were performed by acquiring spectra from 50 randomly selected positions on each substrate (*n* = 50), and the results were reported as mean ± SD, unless otherwise stated. For all UV–vis and SERS data visualizations, the dataset corresponding to the median of each group was selected for representative plotting. Prior to statistical testing, data distributions were assessed for normality by using the Shapiro‐Wilk test and for homogeneity of variances by using Levene's test. As all datasets met the assumptions of normality and equal variance, no data transformation or outlier exclusion was applied. One‐way analysis of variance (ANOVA) used by Tukey's post hoc test was used for multiple group comparisons. For pairwise comparisons, an unpaired two‐tailed Student's *t*‐test was achieved. A significance threshold of *P* < 0.05 was considered statistically significant. The statistical analyses and figure generation were conducted using OriginPro 2022b (OriginLab Corp., Northampton, MA, USA).

## Conflict of Interest

The authors declare no conflict of interest.

## Supporting information



Supporting Information

Supplemental Video 1

Supplemental Video 2

Supplemental Video 3

## Data Availability

Research data are not shared.
