# Supplementary material for: Helicobacter pylori Detection Based on Synergistic Electromagnetic and Chemical Enhancement of Surface‐Enhanced Raman Scattering in 3D Hotspot‐Activated Gold Nanorods/Nano Mica Platelets/ZnO Quantum Dots
Source: Adv Sci (Weinh). 2025 Apr 23;12(28):2503562. doi: 10.1002/advs.202503562 (PMC12302607; doi:10.1002/advs.202503562)
Supplement: Supplementary file 1 — Supporting Information [file ADVS-12-2503562-s001.docx]

Supporting Information

***Helicobacter Pylori* Detection Based on Synergistic Electromagnetic and Chemical Enhancement of Surface-Enhanced Raman Scattering in 3D Hotspot-Activated Gold Nanorods/Nano Mica Platelets/ZnO Quantum Dots**

*Ming-Chang Lu, Yung-Chi Yang, Chia-Jung Lee, and Chih-Wei Chiu**

M.-C. Lu, Y.-C. Yang, C.-W. Chiu

Department of Materials Science and Engineering

National Taiwan University of Science and Technology

Taipei 10607, Taiwan

E-mail: [cwchiu@mail.ntust.edu.tw](mailto:cwchiu@mail.ntust.edu.tw)

C.-J. Lee

Ph.D. Program in Clinical Drug Development of Herbal Medicine

College of Pharmacy

Taipei Medical University

Taipei 11031, Taiwan

**Experimental Section**

**Materials**

HAuCl_4_ (99.9%) and NaBH_4_ (98% purity) were purchased from Echo Chemical Co. (Taiwan). Cetyltrimethylammonium bromide (CTAB) was purchased from Echo Chemical Co. (Taiwan). AgNO_3_ (99.9%) was purchased from Alfa Aesar Co. (USA). Ascorbic acid (99%) was purchased from Acros Organics Co. (USA). H_2_SO_4_ (95–98%) was purchased from Echo Chemical Co. (Taiwan). Synthetic fluorinated mica (SOMASIF ME-100), composed of Si (26.5 wt%), Mg (15.6 wt%), F (8.8 wt%), Na (4.1 wt%), Al (0.2 wt%), and Fe (0.1 wt%), was purchased from CO-OP Chemical Co. (Japan). Jeffamine-T403 was purchased from Huntsman Chemical Co. (USA). Bisphenol A epoxy resin BE-188 was purchased from Echo Chemical Co. (Taiwan). HCl (37%) was purchased from Echo Chemical Co. (Taiwan). NaOH was purchased from Macron Fine Chemicals (Avantor Performance Materials, Center Valley, PA, USA). Ethanol (95%) was purchased from Echo Chemical Co. (Taiwan). Zn(CH_3_COO)_2_·2H_2_O (99%) was purchased from Showa Chemical Industry Co. (Japan). KOH (>85%) was purchased from Shimakyu Pure Chemicals Co. (Japan). Methanol (99%) was purchased from Echo Chemical Co. (Taiwan). Tetraethoxysilane (98%) was purchased from Acros Organics Co. (USA). Adenine (99.9%) and cytosine (99%) were purchased from Sigma-Aldrich Chemical Co. (USA). Thymine (99%) and guanine (99%) were purchased from Acros Organics Co. (USA). *Helicobacter pylori* (ATCC 43504) was purchased from Academia Sinica. *Staphylococcus aureus* (ATCC 25923) was purchased from the Bioresource Collection and Research Center (Taiwan). *Escherichia coli* (ATCC 25922) was purchased from the American Type Culture Collection (USA). Blood agar plates (TSA with 5% sheep blood) were purchased from Creative Life Science Co. (Taiwan). Ultrapure water (resistivity = 18.2 MΩ cm at 25 °C) was obtained using a Milli-Q water purification system. All reagents were utilized in their original state without any additional purification or treatment. All glassware was subjected to a thorough cleaning, which involved washing with *aqua regia*, rinsing with water, and drying.

**Preparation of Gold Nanorods (AuNRs)**

AuNRs were prepared using a modification of a previously reported seed-mediated method,^[1]^ with the nanorod aspect ratio controlled by varying the concentration of Ag^+^. HAuCl_4_ (0.024 m, 0.1 mL) and CTAB (0.1 m, 7.5 mL) solutions were mixed, and the mixture was supplemented with NaBH_4_ (0.01 m, 0.6 mL) and DI water (1.8 mL) upon cooling in an ice bath and stirred for 2 min to form gold particles with a diameter of less than 2.5 nm. The seed solution underwent a rapid color change from orange to bronze and was left at room temperature for 2 h to stabilize. Subsequently, this solution (0.1 mL) was diluted with aqueous CTAB (0.1 m, 0.9 mL) and sequentially supplemented with CTAB (0.1 m, 10 mL), HAuCl_4_ (0.024 m, 0.2 mL), AgNO_3_ (0.01 m, 0.00–0.10 mL), and H_2_SO_4_ (0.5 m, 0.2 mL) solutions. Upon the following slow introduction of ascorbic acid (0.1 m, 0.08 mL), the solution became colorless. Next, the gold seed solution (0.024 mL) was introduced, and the mixture was gently stirred for 1 min and allowed to stand at room temperature for 2 h to form AuNRs. The resulting brown solution was centrifuged at 6,000 rpm for 30 min, and the pellet was washed at least twice with DI water to remove residual CTAB. The thus purified AuNR solution was analyzed by UV–vis spectroscopy, transmission electron microscopy (TEM), and surface-enhanced Raman scattering (SERS).

**Preparation of AuNRs/Nano Mica Platelets (NMPs)**

NMPs were prepared in accordance with a previously reported procedure (**Figure S21**).^[2]^ Polyetheramine (Jeffamine-T403) and epoxy resin (BE-188) were dissolved in ethanol at a 2:1 molar ratio. The solution was stirred for 6 h at 60 °C until a light yellow color was observed, which indicated reaction completion and delaminating agent (T403AEO) formation. Subsequently, mica (1.0 g, 1 wt%) was expanded at 80 °C for 1 h. A mixture of T403AEO (4.8 g) and HCl (0.75 g) was ultrasonicated for 30 min (to facilitate the protonation of the amine group and form a quaternary ammonium salt) and added to the expanded mica slurry, and the resulting dispersion was stirred for 5 h to induce mica delamination. The mixture was purified through extraction with dilute ethanolic NaOH, and the solid was redispersed in deionized water to form a 1 wt% NMPs dispersion. Atomic force microscopy (AFM) revealed that the thickness of mica particles was approximately 13.41 nm due to layer stacking (**Figure S22**). As shown in **Figure S23**, the thickness of monolithic NMPs was ~2.16 nm. The observed variation in thickness confirmed the efficacy of the delamination and dispersion effects. The occurrence of delamination was confirmed by X-ray diffraction (XRD) analysis (**Figure S24**). The original *d*-spacing of mica was determined as 12.7 Å using the peak at 2*θ* = 7°. The addition of the delaminating agent for cation exchange and polymerization between the layers resulted in the disruption of van der Waals forces and, hence, clay delamination. This process was confirmed by the absence of wrap-around XRD peaks, which indicated that the mica had delaminated into monolithic NMPs. Mixtures of NMPs and HAuCl_4_ (0.024 m, 0.2 mL) with different weight ratios were supplemented with the gold seed solution (0.1 mL) and CTAB (0.1 m, 10.9 mL) and stirred for 1 h. Subsequently, AgNO_3_ (0.01 m, 0.08 mL) and H_2_SO_4_ (0.5 m, 0.2 mL) were added, and the mixture was dropwise supplemented with ascorbic acid (0.1 m, 0.08 mL). Then, the gold seed solution (0.024 mL) was added, and the dispersion was thoroughly mixed for ~1 min and allowed to stand for ~2 h. The resulting nanohybrid (AuNRs/NMPs) solution was centrifuged at 6,000 rpm for 30 min, and the pellet was washed at least twice with deionized water to remove residual CTAB. To confirm the effective suppression of background interference, control SERS measurements were performed on blank substrates without target molecules. The comparative SERS spectra of CTAB (0.1 m), NMPs, and AuNRs/NMPs indicate minimal background contributions in the spectral regions of interest (**Figure S25**). The characteristic peaks of CTAB are nearly absent in the AuNRs/NMPs spectrum, verifying that surfactant-induced Raman interference was effectively eliminated through our purification process. The purified solution of AuNRs/NMPs was analyzed using UV–vis spectroscopy, zeta potential measurements, TEM, energy-dispersive X-ray spectroscopy (EDS), and SERS.

**Preparation of AuNRs/NMPs/ZnO QDs**

ZnO QDs were prepared using a sol-gel synthesis method.^[3]^ A methanolic solution of KOH (1 m, 25 mL) solution was slowly added to that of Zn(CH_3_COO)_2_·2H_2_O (0.1 m, 12.5 mL) at room temperature (25 °C), and the mixture was stirred for 1 h at 1,000 rpm. The presence of ZnO particles was confirmed by the observation of bright yellow fluorescence under UV light. Subsequently, tetraethoxysilane (0.125 mL) was added to regulate particle growth, and deionized water (0.25 mL) was introduced into the resulting gel solution to facilitate a sol-gel reaction on the particle surface. After purification by at least two-fold centrifugation with methanol and deionized water to remove residual reactive molecules, the gel was redissolved in deionized water to form a solution of ZnO QDs. Given the abundance of hydroxyl groups on the silicate nanoclay surface, AuNRs/NMPs with varying weight ratios were physically mixed with ZnO QDs for 15 min to facilitate the stabilization of the two materials through hydrogen bonding and form AuNRs/NMPs/ZnO QDs, which were characterized by TEM, EDS, and SERS.

**Culturing of Bacteria and Preparation of SERS Substrate**

Prior to bacterial culturing, all glassware was sterilized via autoclaving at an elevated pressure and temperature (~120 °C, 50 min). For bacterial growth, broth and Lysogeny broth (LB) were prepared in separate 250 mL serum bottles. *S. aureus* (ATCC 25923) was inoculated into 10 mL of broth, while *E. coli* (ATCC 25922) was inoculated into 10 mL of LB using an inoculating loop. Both bacterial cultures were incubated at 37 °C for 18–24 h to ensure optimal growth. The broth was prepared in a 250 mL serum bottle, and *H. pylori* (ATCC 43504) was transferred into the broth (2 mL) using an inoculation ring. Subsequently, the sample was smeared in blood culture medium and cultured in an anaerobic culture tank with microaerobic gas packs at 37 °C for 7 days. The colonies were collected and transferred to a microcentrifuge tube containing 2 mL of the broth using an inoculating loop. The collected bacterial suspension was divided into two 1 mL portions, one of which was used to spread the blood culture medium for the subsequent round of culturing to ensure the continuity and stability of the culture, while the other was subjected to UV–vis analysis and OD_600_-adjusted to 0.6. Thereafter, the growing colonies were quantified. The bacterial solution was centrifuged at 6,000 rpm for 3 min. The upper layer was removed and washed three times with phosphate-buffered saline and then prepared for SERS. BALB/cByJNarl male mice (6 weeks old, 10 mice in total) procured from the National Laboratory Animal Center (Taipei, Taiwan) were maintained in a constant-temperature environment at 24 °C and subjected to a 12 h light/12 h dark cycle (light hours from 06:00 to 18:00), with food and water provided *ad libitum*. The animal experimental protocols had received approval from the Institutional Animal Care and Use Committee of Taipei Medical University (LAC-2020-0265). Subsequently, solutions of AuNRs, AuNRs/NMPs, and AuNRs/NMPs/ZnO QDs (10 μL) were drop-coated onto clean aluminum sheets with dimensions of 10 mm × 10 mm and oven-heated at 80 °C. Analyte solutions were prepared at concentrations of 10^−4^–10^−11^ m for adenine, 10^−4^ m for thymine, 10^−4^ m for guanine, and 10^−4^ m for cytosine (volume = 10 μL). The *H. pylori* solution (10 μL) or mouse fecal matter containing *H. pylori* (in a 1:1 weight ratio) were combined with the SERS substrate in a nutrient-free environment and subsequently dried for Raman detection.

**Characterization**

The thickness and phase images of mica and delaminated clays (NMPs) were examined using AFM (Bruker Dimension Icon) in tapping mode. The XRD analyzer (D2 PHASER XE-T) was equipped with a Cu *K*_α_ radiation source (*λ* = 1.54056 Å) and operated using a scan (2*θ*) range of 3°–20° to observe the variations within the clay layers. UV–vis spectra were recorded using a Shimadzu UV-2450 instrument (Kyoto, Japan). For TEM imaging (JEOL JEM-2100), a 10 μL droplet of the SERS substrate suspension was placed on a copper mesh and allowed to dry at 80 °C for 24 h. Confocal micro-Raman spectroscopy (Horiba-LABRAM HR800, 785 nm laser) was used to for SERS analysis in the 400–4000 cm^−1^ range (exposure = 10 s, power = 20 mW). The excitation light was focused on an excitation area of ~4 μm^2^ using a 50× objective lens. Finite-difference time-domain (FDTD) simulations of the electric field distribution were performed using Lumerical FDTD Solutions software, with the excitation laser wavelength set to 785 nm. Zeta potential analysis was conducted using a Zetasizer Nano ZS90 instrument. Samples for high-resolution field-emission scanning electron microscopy (JSM-6500F) were prepared by drop-coating clean glass surfaces with the desired dispersions followed by oven-drying at 80 °C for 2 h, affixing to a conductive carbon adhesive, and coating with a thin layer of Pt.


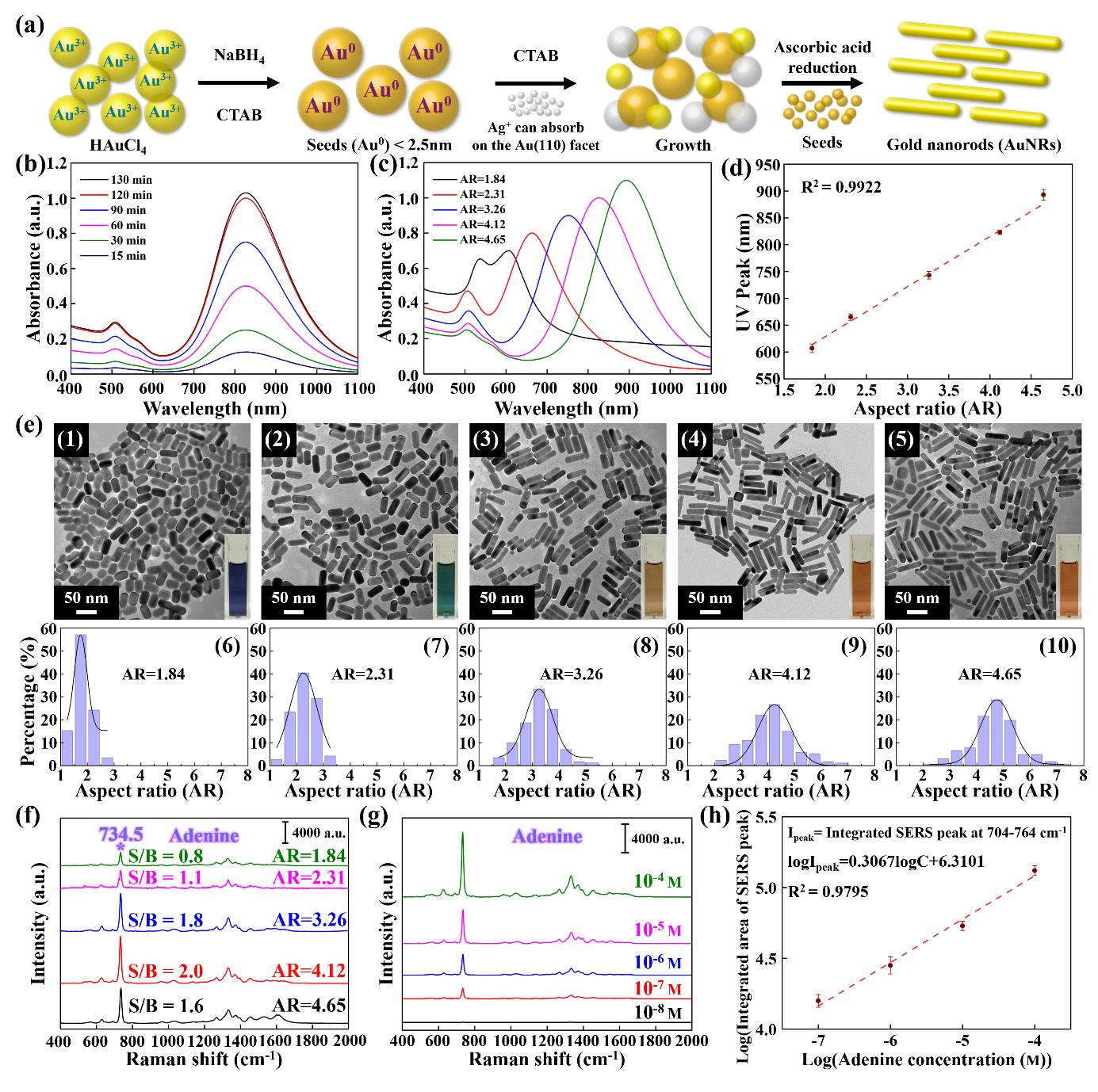


**Figure S1.** (a) Schematic synthesis of AuNRs. (b) UV–vis spectra of AuNRs collected at different times during synthesis (*n* = 10). The spectrum corresponding to the median set was selected for representative plotting. (c) UV–vis spectra of AuNRs with varying aspect ratios (*n* = 10). (d) Correlation between the absorption peak position and mean aspect ratio of AuNRs. (e) Transmission electron microscopy images of AuNRs with mean aspect ratios of (1) 1.84, (2) 2.31, (3) 3.26, (4) 4.12, and (5) 4.65, with the corresponding distributions determined using the Image J software provided in (6)–(10) (insets are photographs of the related dispersions). (f) SERS responses of AuNRs with different aspect ratios to adenine (10^−4^ m). Spectra were averaged over 50 randomly selected positions (*n* = 50), with the median set displayed. (g) SERS responses of AuNRs with an aspect ratio of 4.12 to different adenine concentrations (*n* = 50). (h) Linear fit of the log(integrated intensity in the range of 704–764 cm^−1^)–log(adenine concentration) plot.


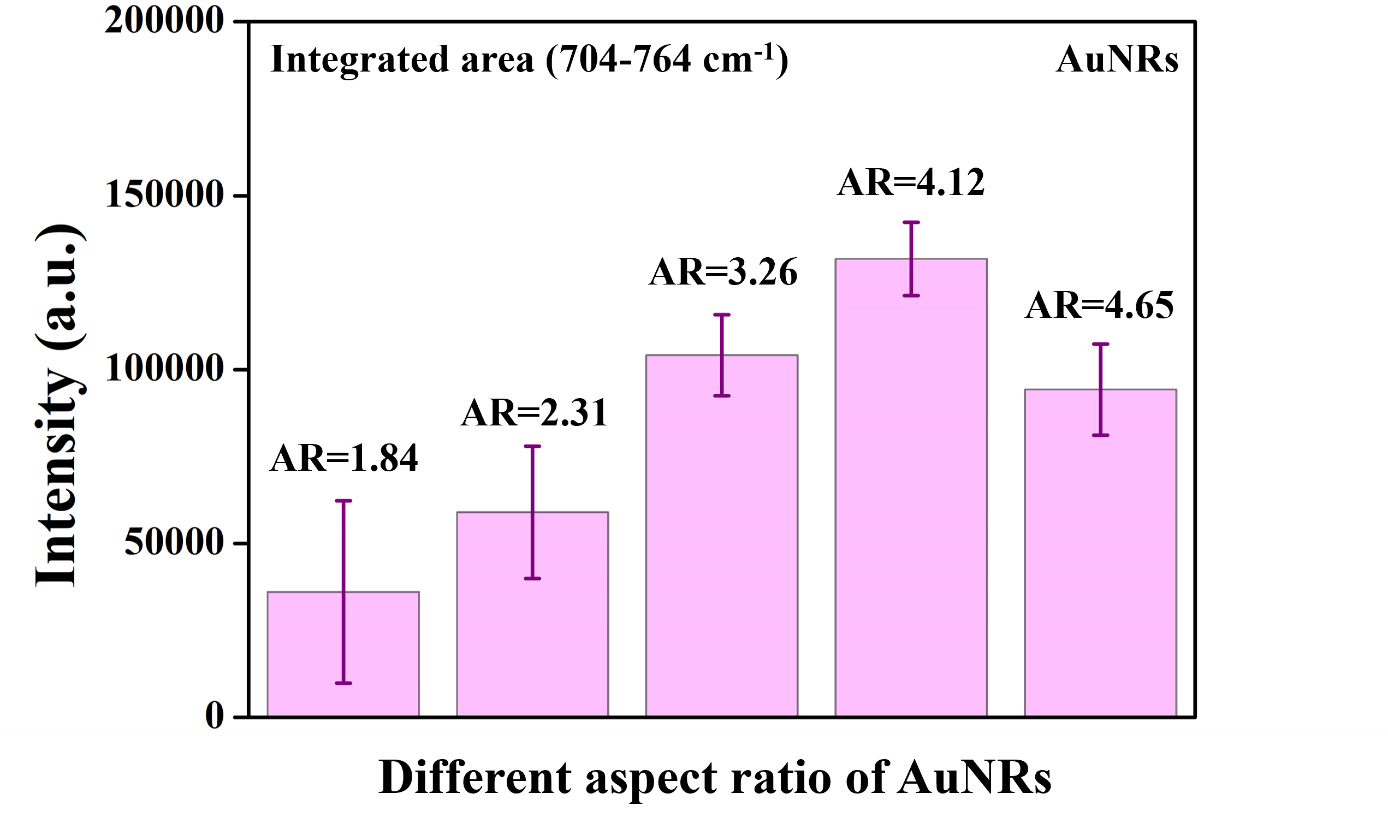


**Figure S2.** Integrated intensities of the SERS responses (704–764 cm^−1^) of AuNRs with various aspect ratios to adenine (10^−4^ m; *n* = 50).


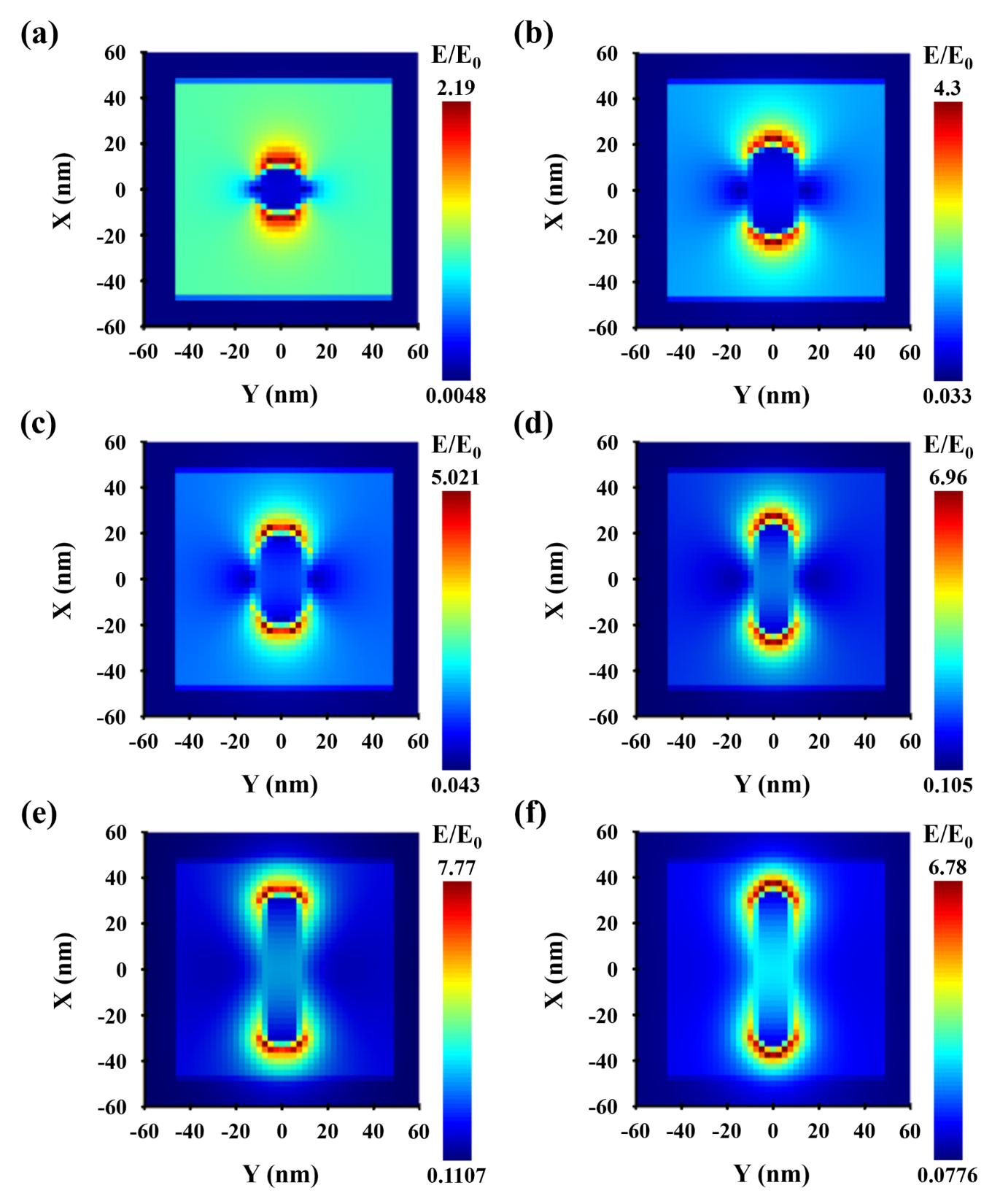


**Figure S3.** FDTD simulations of the electric field distribution (y-x plane) for AuNRs with mean aspect ratios of (a) 1, (b) 1.84, (c) 2.31, (d) 3.26, (e) 4.12, and (f) 4.65 under 785 nm laser excitation.


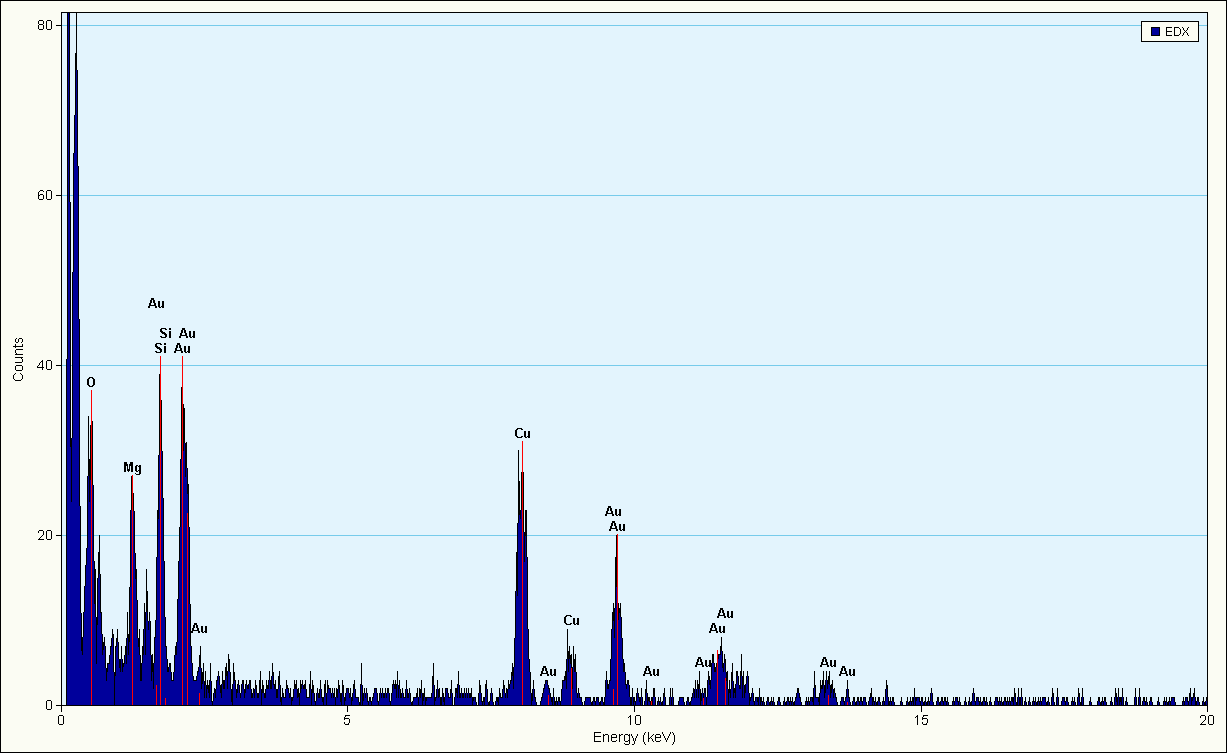


**Figure S4.** EDS profile of AuNRs/NMPs.


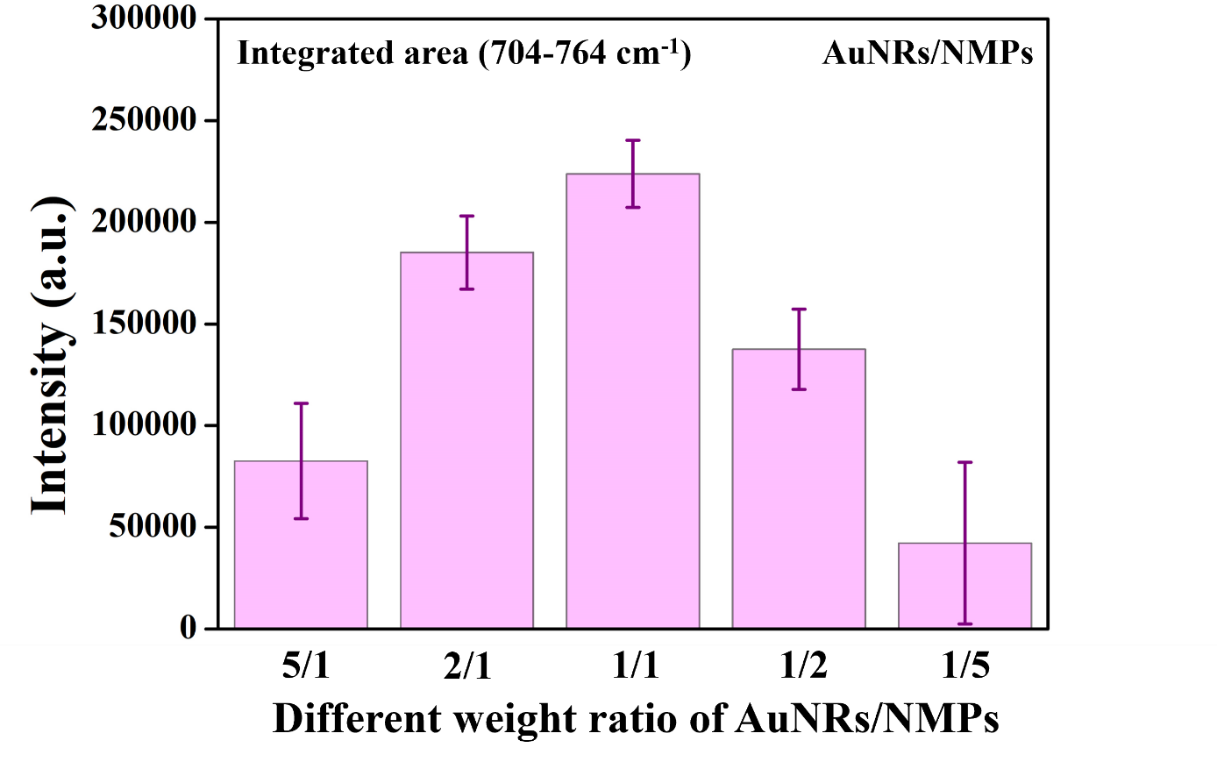


**Figure S5.** Integrated intensities of the SERS responses (704–764 cm^−1^) of AuNRs/NMPs with various weight ratios to adenine (10^−4^ m; *n* = 50).


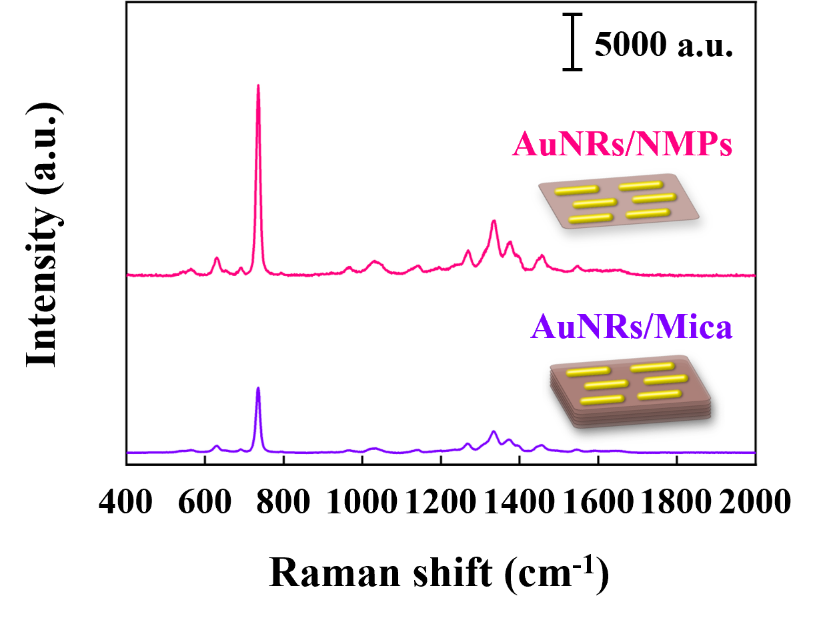


**Figure S6.** SERS responses for adenine detection (10^−4^ m) using (a) AuNRs/NMPs (1/1, w/w) and (b) AuNRs/Mica. Spectra were averaged over 50 randomly selected positions (*n* = 50), with the median set displayed.


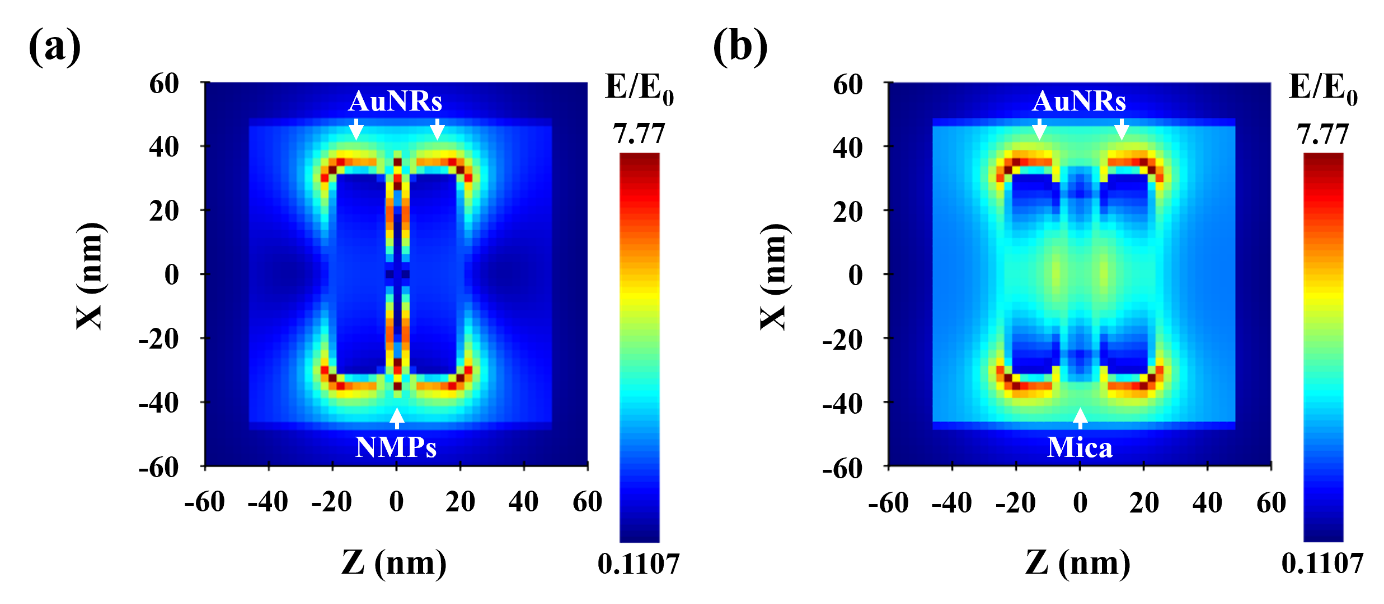


**Figure S7.** FDTD simulations of the electric field distribution (z-x plane) for (a) AuNRs/NMPs and (b) AuNRs/Mica under 785 nm laser excitation.


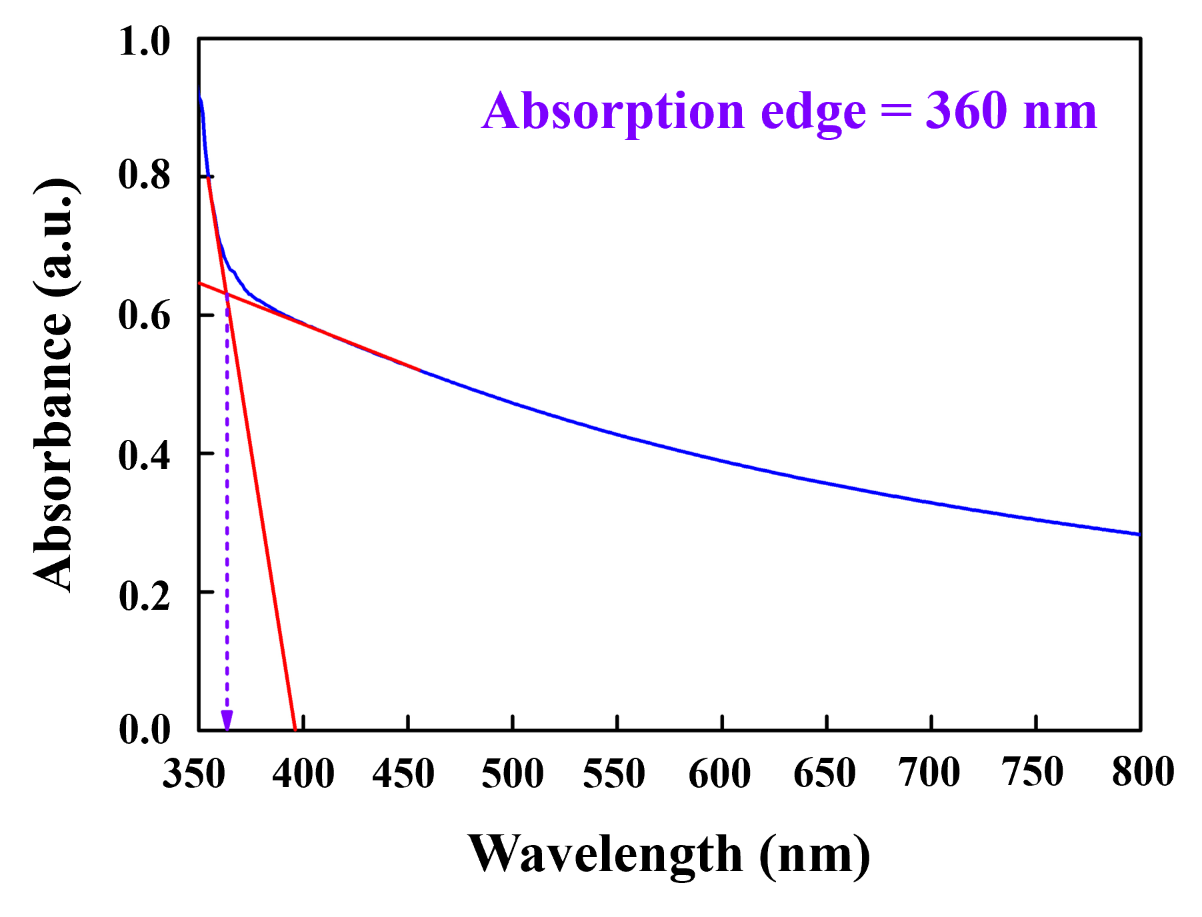


**Figure S8.** UV–vis spectrum of ZnO QDs showing an absorption edge at 360 nm.


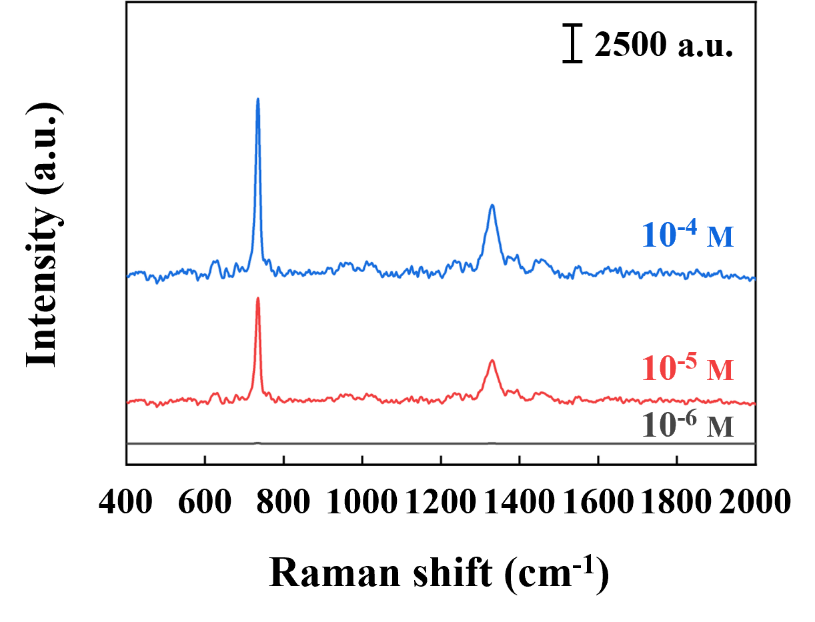


**Figure S9.** SERS responses of ZnO QDs to different adenine concentrations. Spectra were averaged over 50 randomly selected positions (*n* = 50), with the median set displayed.


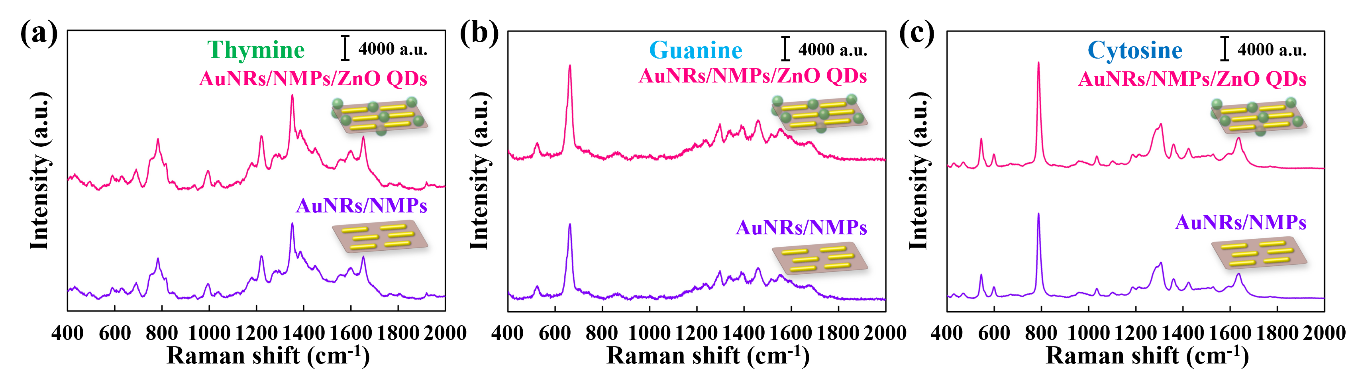


**Figure S10.** SERS responses of AuNRs/NMPs/ZnO QDs (1/1/2, w/w) and AuNRs/NMPs to (a) thymine, (b) guanine, and (c) cytosine (all 10^−4^ m). Spectra were averaged over 50 randomly selected positions (*n* = 50), with the median set displayed.


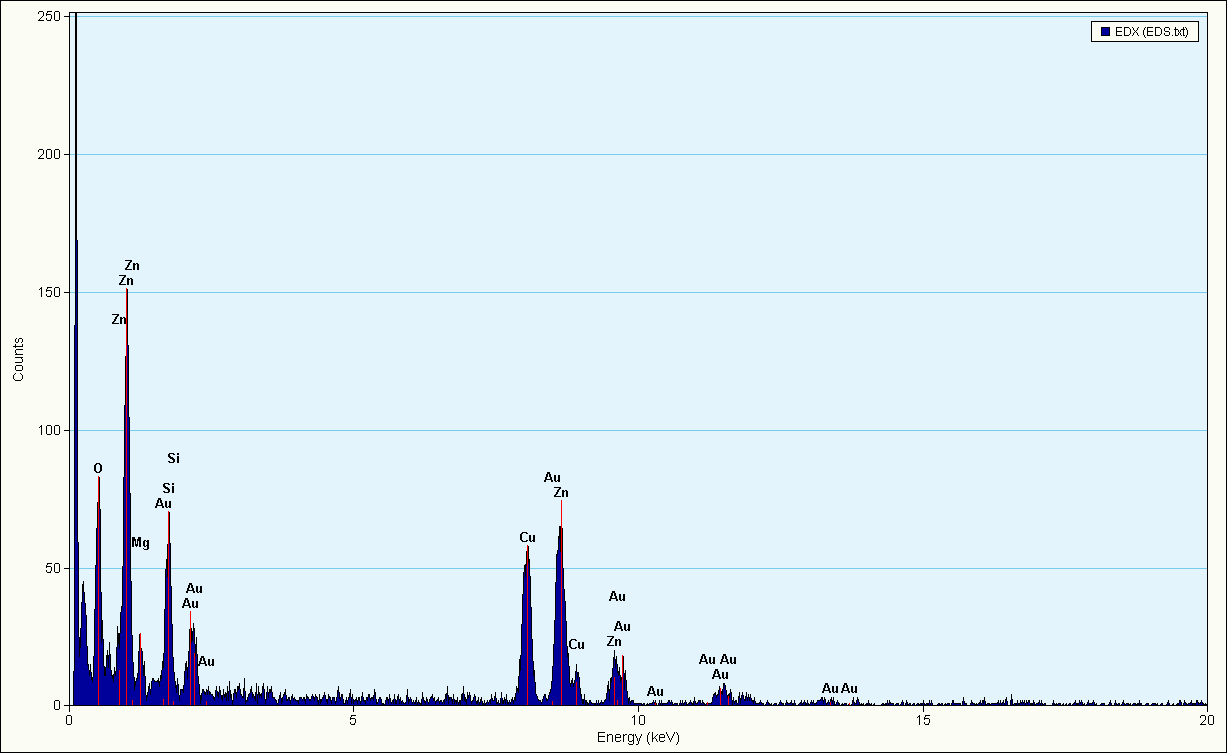


**Figure S11.** EDS profile of AuNRs/NMPs/ZnO QDs.


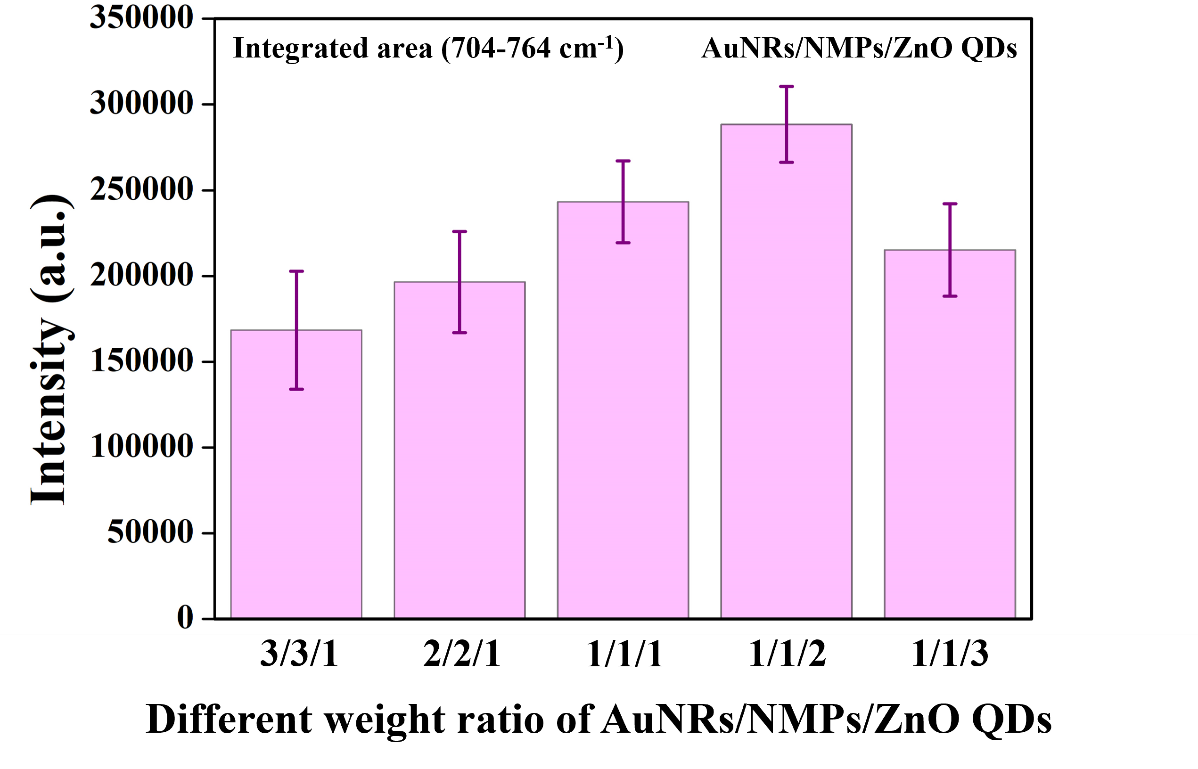


**Figure S12.** Integrated intensities of the SERS responses (704–764 cm^−1^) of AuNRs/NMPs/ZnO QDs with various weight ratios to adenine (10^−4^ m; *n* = 50).


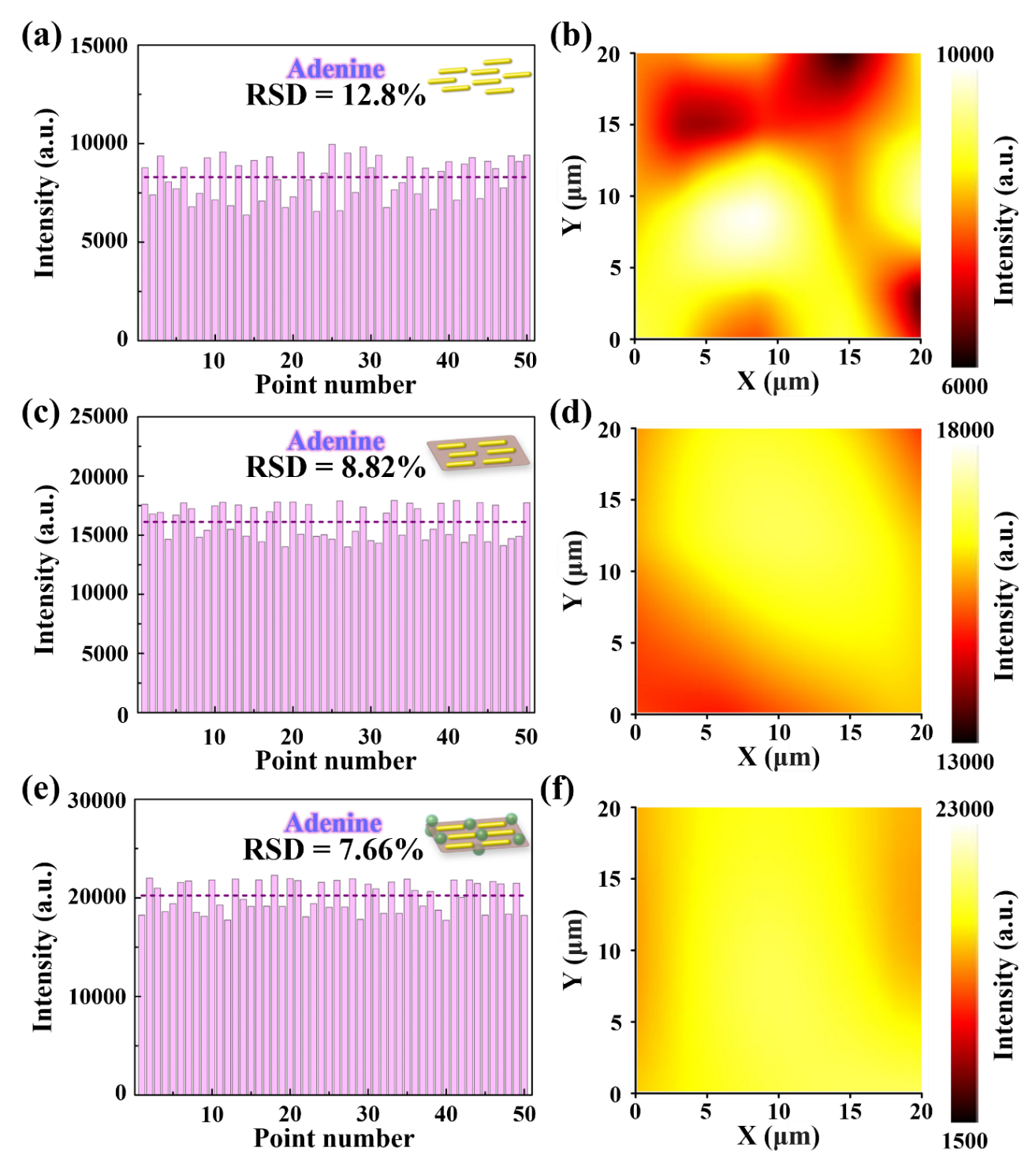


**Figure S13.** (a) Raman signal intensities of adenine (10^−4^ m, 734.5 cm^−1^) detected at 50 random points on the surface of AuNRs with an average aspect ratio of 4.12. (b) Distribution of the Raman signal intensity of adenine (10^−4^ m, 734.5 cm^−1^) over a 400 μm^2^ area on the AuNR surface. (c) Raman signal intensities of adenine (10^−4^ m, 734.5 cm^−1^) detected at 50 random points on the surface of AuNRs/NMPs (1/1, w/w). (d) Distribution of the Raman signal intensity of adenine (10^−4^ m, 734.5 cm^−1^) over a 400 μm^2^ area on the surface of AuNRs/NMPs. (e) Raman signal intensities of adenine (10^−4^ m, 734.5 cm^−1^) detected at 50 random points on the surface of AuNRs/NMPs/ZnO QDs (1/1/2, w/w). (f) Distribution of the Raman signal intensity of adenine (10^−4^ m, 734.5 cm^−1^) over a 400 μm^2^ area on the surface of AuNRs/NMPs/ZnO QDs.


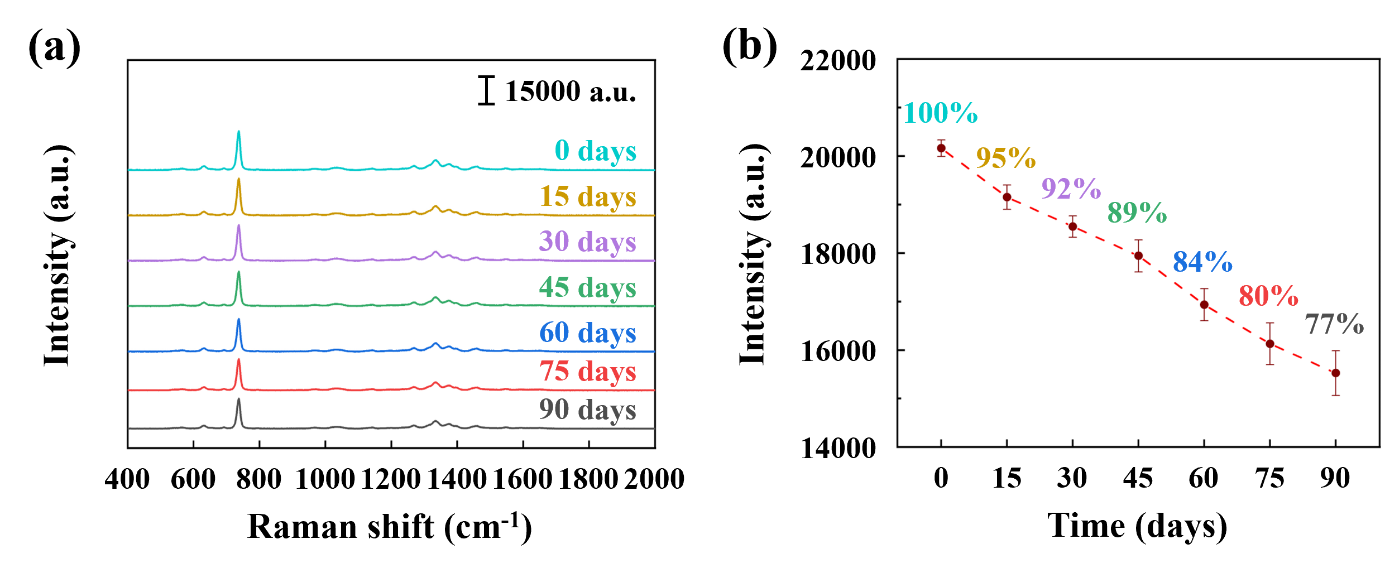


**Figure S14.** (a) SERS responses of adenine (10^−4^ m) detected using AuNRs/NMPs/ZnO QDs after different storage durations. Spectra were averaged over 50 randomly selected positions (*n* = 50), with the median set displayed. (b) Retention of the Raman signal intensity at 734.5 cm^−1^ over 90 days.


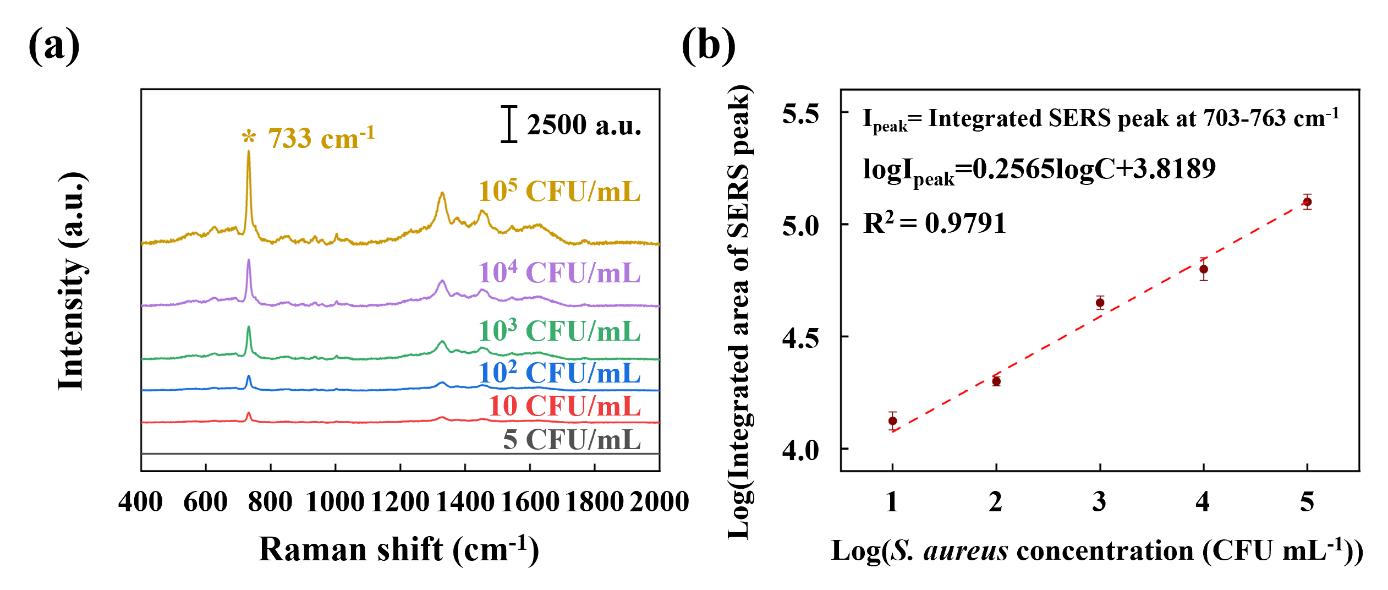


**Figure S15.** (a) SERS responses of *S. aureus* at various concentrations using AuNRs/NMPs/ZnO QDs. Spectra were averaged over 50 randomly selected positions (*n* = 50), with the median set displayed. (b) Linear fit of the log(integrated intensity in the range of 703–763 cm^−1^)– log(*S. aureus* concentration) plot.


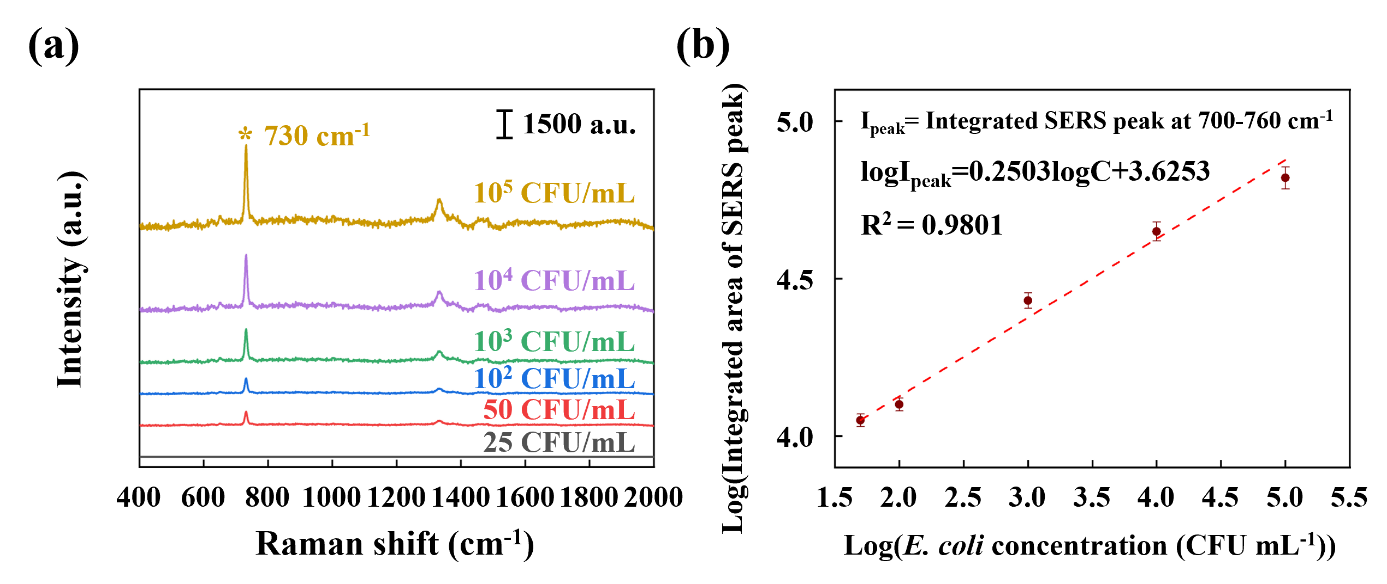


**Figure S16.** (a) SERS responses of *E. coli* at various concentrations using AuNRs/NMPs/ZnO QDs. Spectra were averaged over 50 randomly selected positions (*n* = 50), with the median set displayed. (b) Linear fit of the log(integrated intensity in the range of 700–760 cm^−1^)– log(*E. coli* concentration) plot.


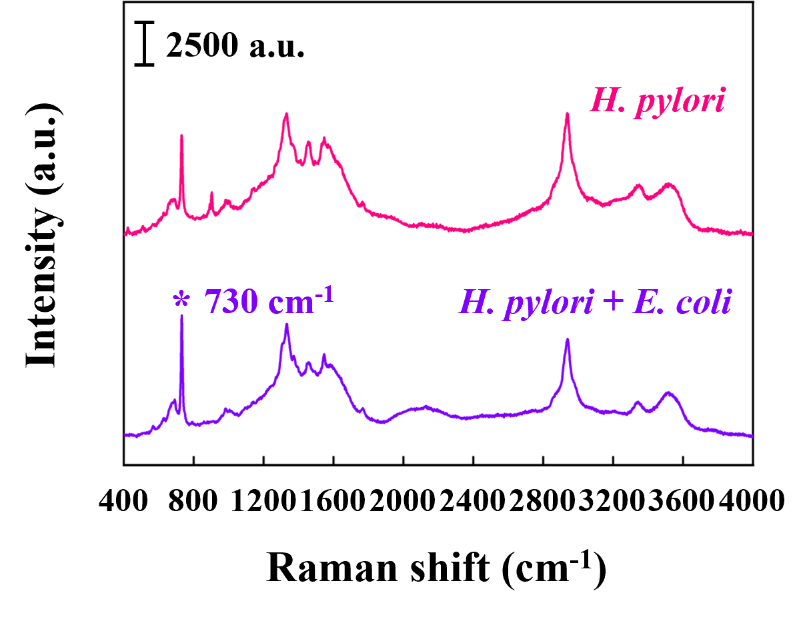


**Figure S17.** SERS responses of *H. pylori* (1.8 × 10^4^ CFU mL^−1^) alone and in a mixed sample with *E. coli* using AuNRs/NMPs/ZnO QDs. Spectra were averaged over 50 randomly selected positions (*n* = 50), with the median set displayed.


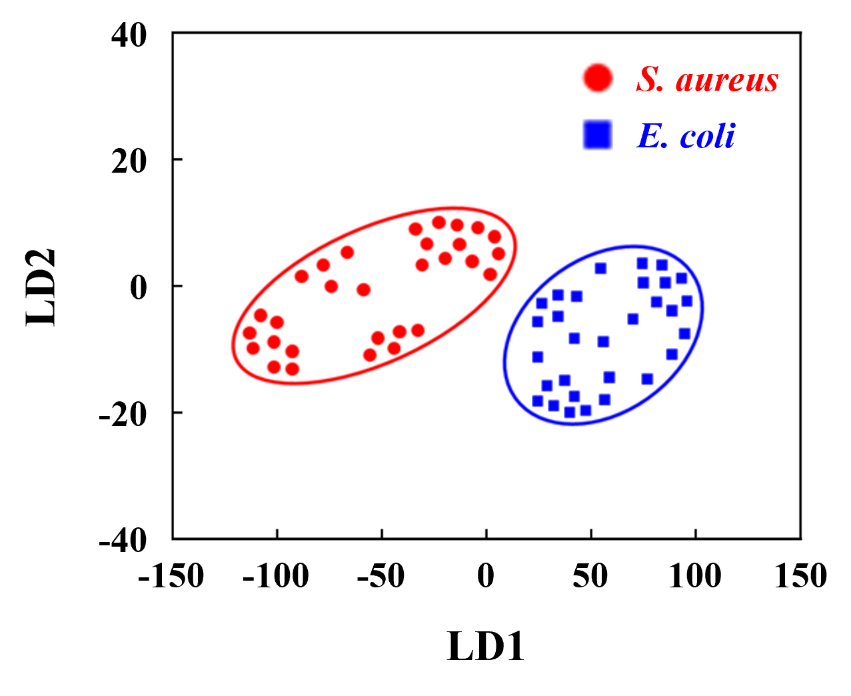


**Figure S18.** PCA plot illustrating the spectral separation between *S. aureus* (red line) and *E. coli* (blue line) based on SERS data (*n* = 30).


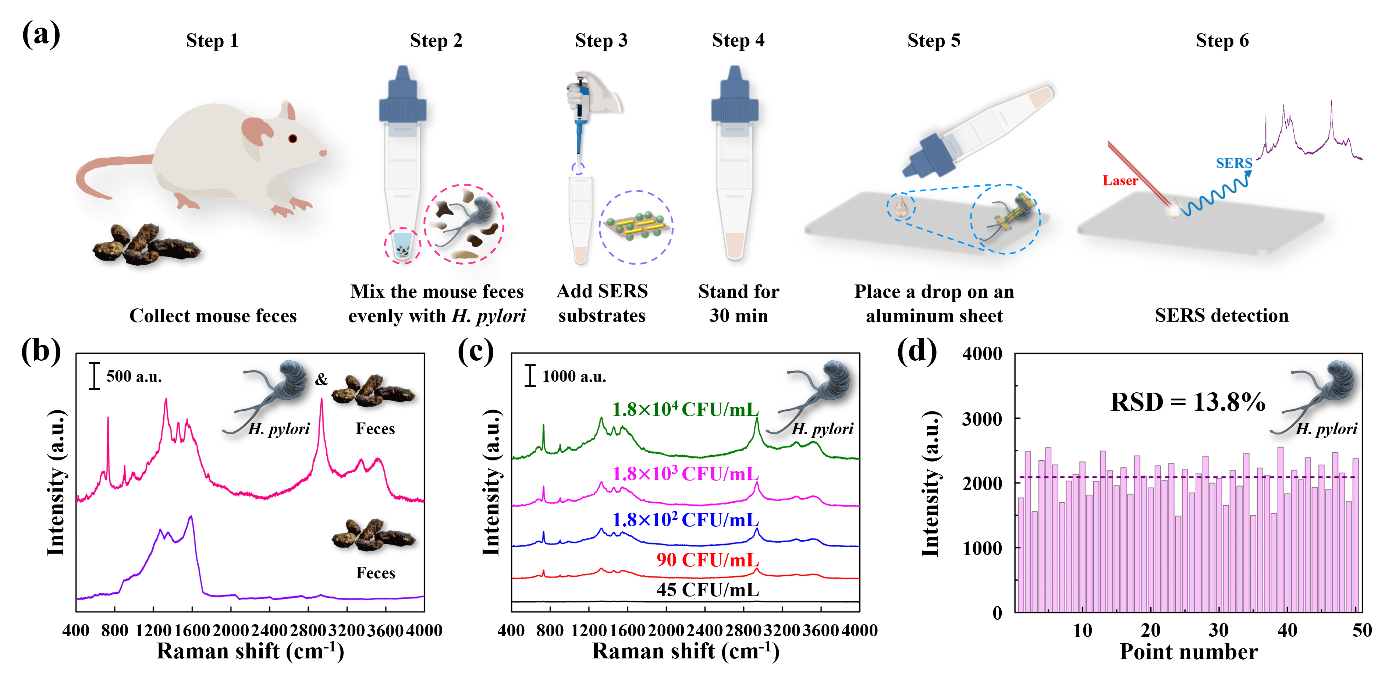


**Figure S19.** (a) Schematic SERS-based detection of *H. pylori* in mouse fecal samples by AuNRs/NMPs/ZnO QDs. (b) SERS responses of AuNRs/NMPs/ZnO QDs (1/1/2, w/w) to a mixture of *H. pylori* and mouse feces (1.8 × 10^4^ CFU mL^−1^). Spectra were averaged over 50 randomly selected positions (*n* = 50), with the median set displayed. (c) SERS responses of AuNRs/NMPs/ZnO QDs (1/1/2, w/w) to *H. pylori*–containing mouse fecal solutions with different concentrations (*n* = 50). (d) Intensities of the SERS responses of AuNRs/NMPs/ZnO QDs (1/1/2, w/w) determined at randomly selected points (10 mouse fecal samples with *H. pylori* (1.8 × 10⁴ CFU mL^−1^), 5 points per sample).


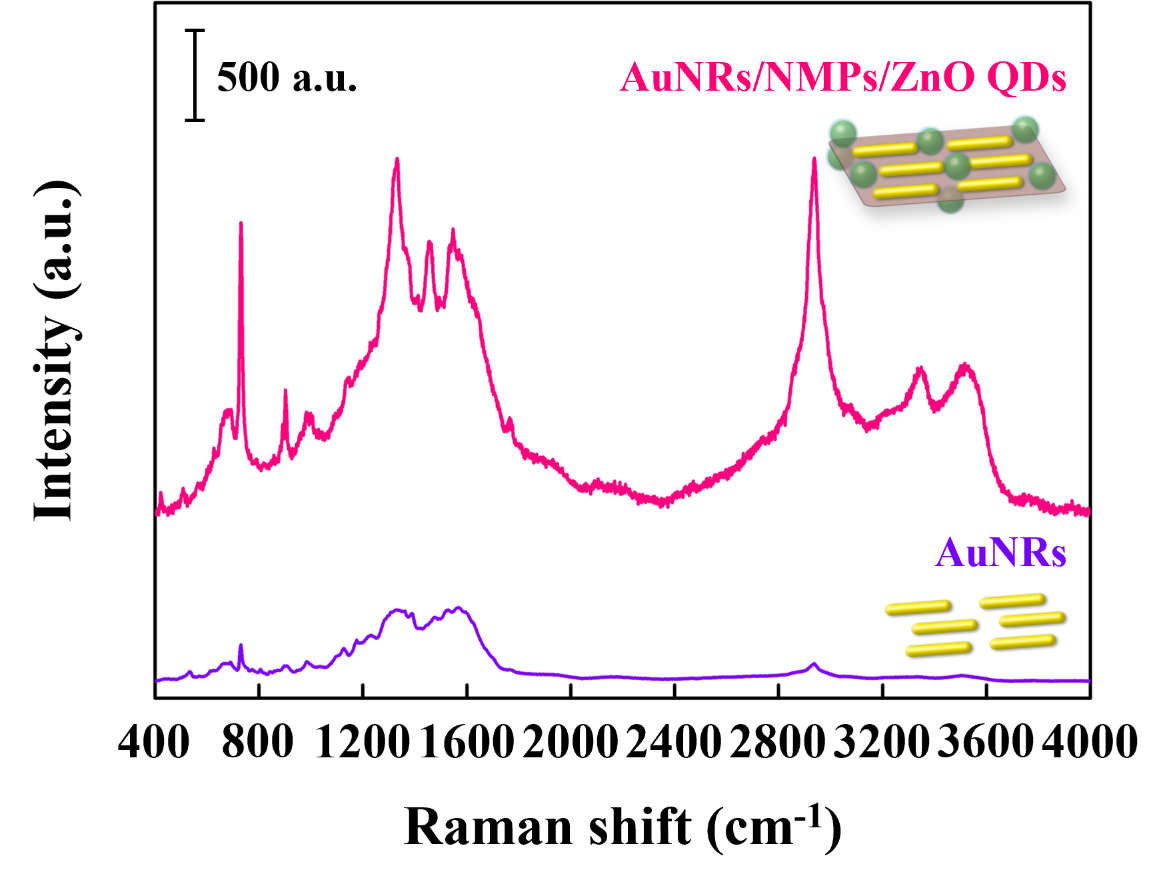


**Figure S20.** SERS responses of AuNRs/NMPs/ZnO QDs (1/1/2, w/w/w) and AuNRs to a mouse fecal solution containing *H. pylori* (1.8 × 10^4^ CFU mL^−1^). Spectra were averaged over 50 randomly selected positions (*n* = 50), with the median set displayed.


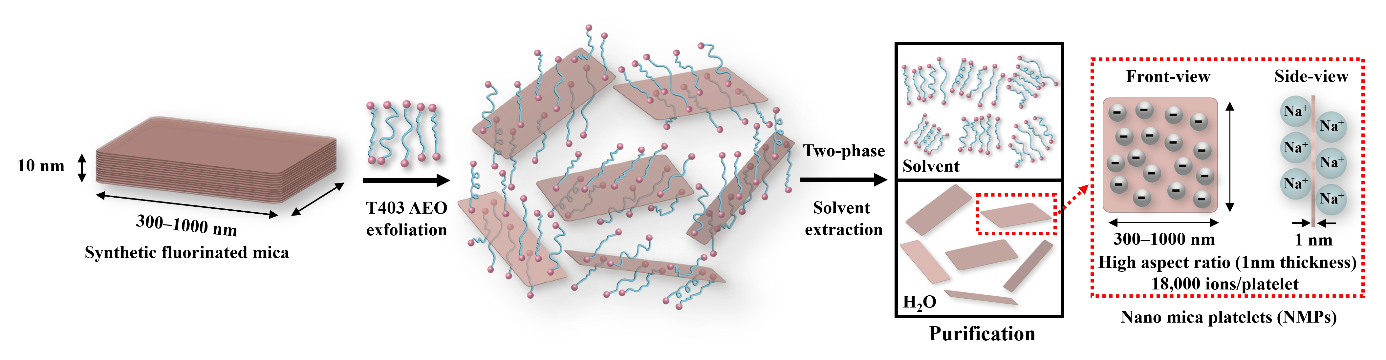


**Figure S21.** Schematic synthesis of NMPs based on one-step delamination.


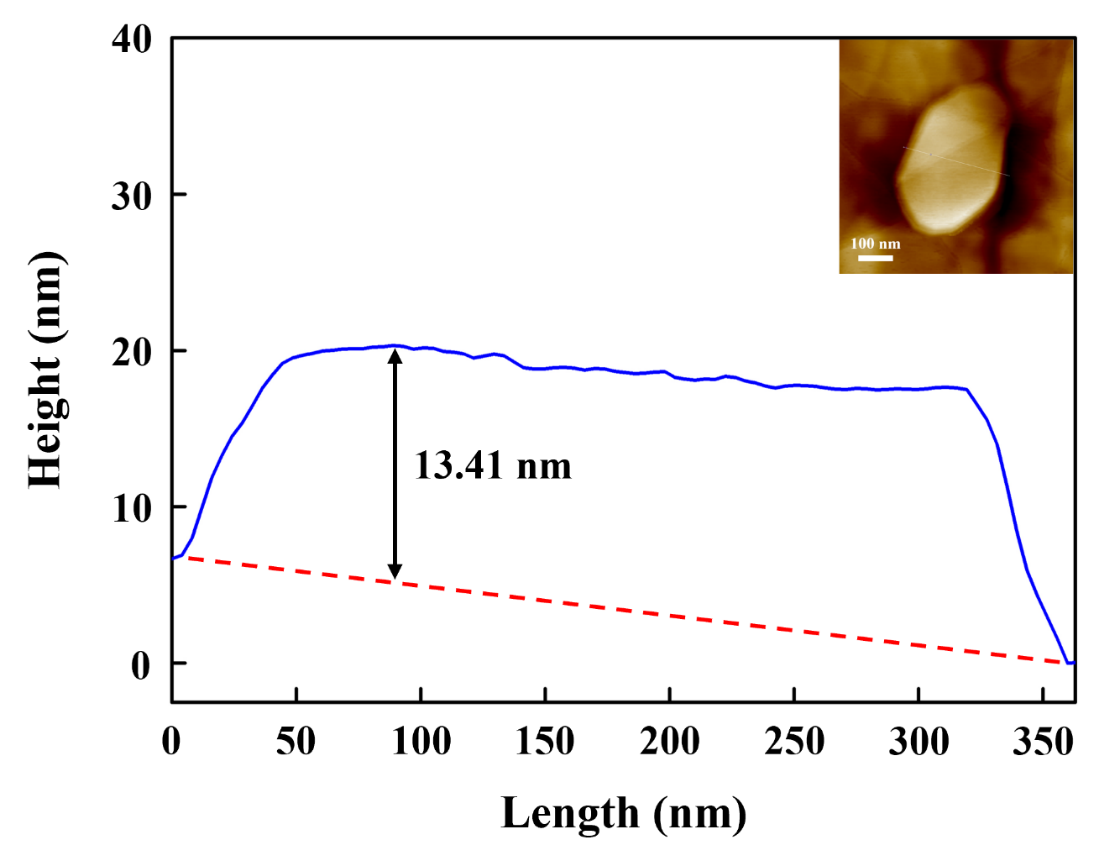


**Figure S22.** Height distribution map of original mica illustrating the presence of a multilayered stacked structure with an approximate thickness of 13.41 nm. Inset depicts an AFM image of an original mica particle.


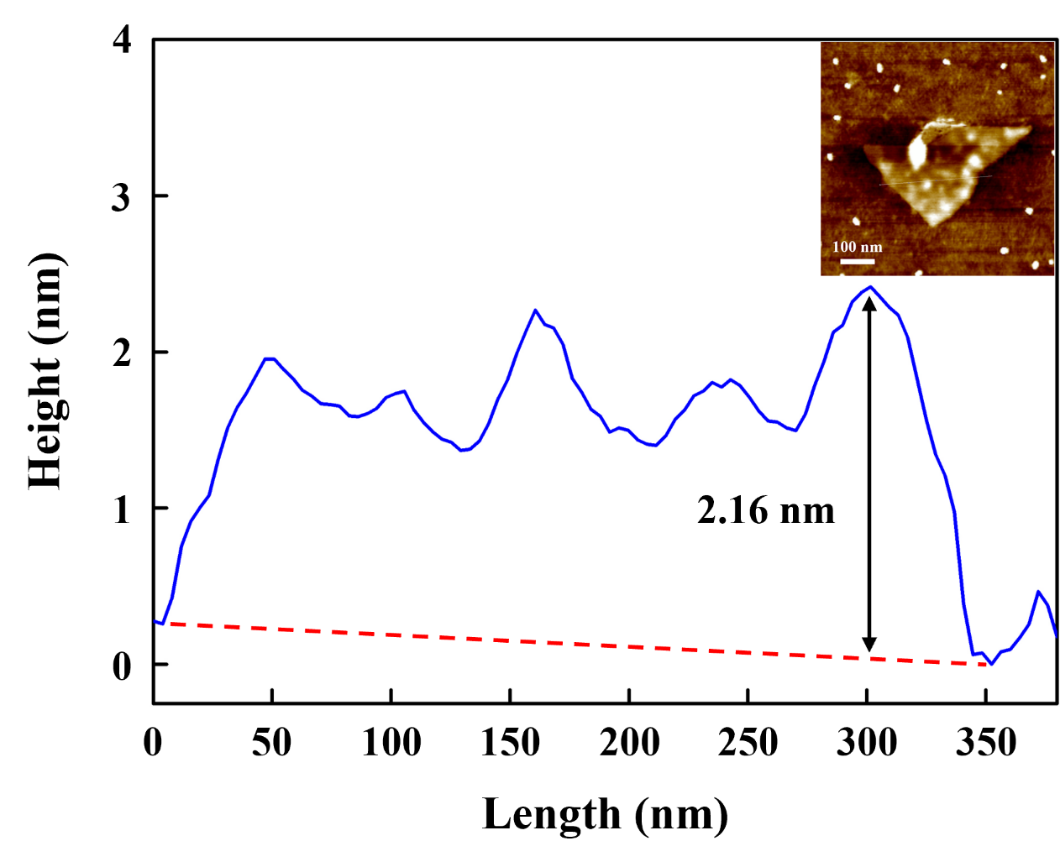


**Figure S23.** Height distribution map of NMPs with a thickness of ~2.16 nm. Inset depicts an AFM image of a single NMP.


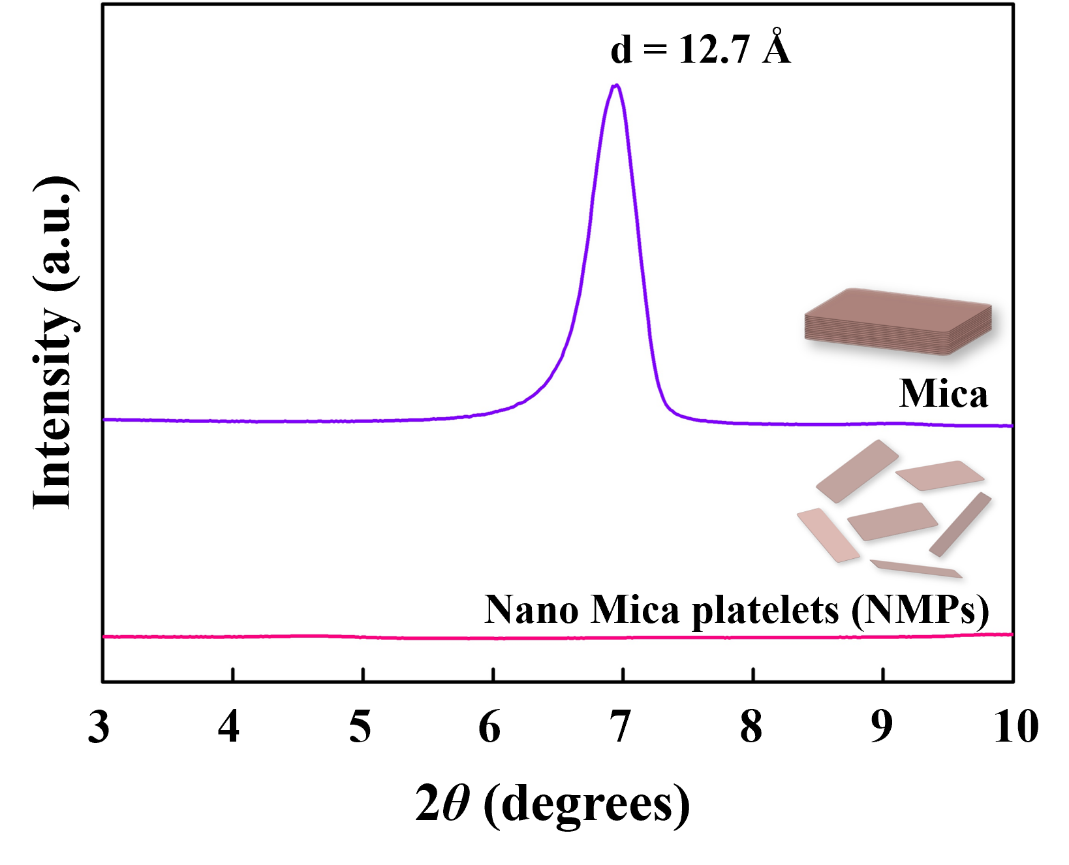


**Figure S24.** XRD patterns of the original mica and NMPs produced by its delamination.


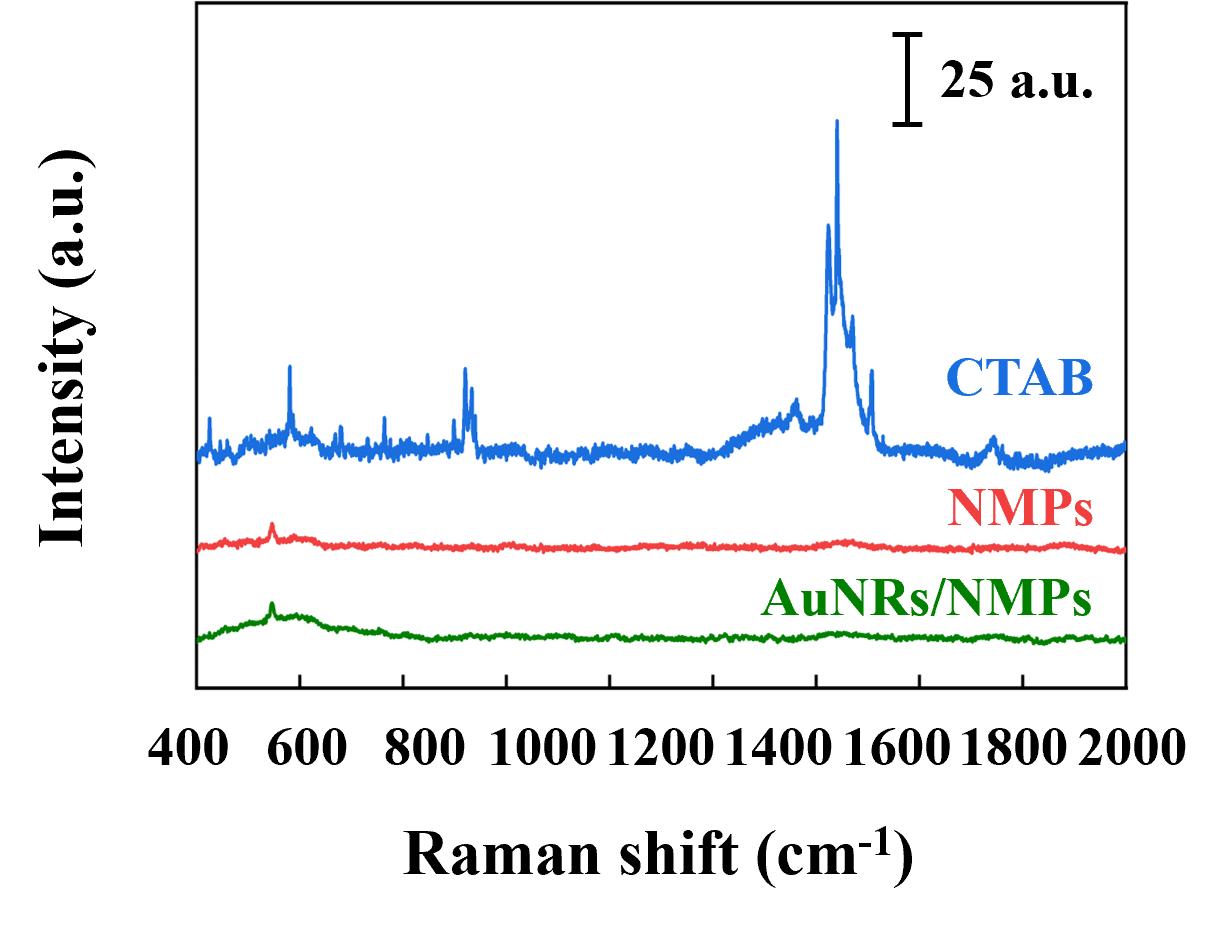


**Figure S25.** SERS responses of CTAB (0.1 m), NMPs, and AuNRs/NMPs without target molecules. Spectra were averaged over 10 randomly selected positions (*n* = 10), with the median set displayed.

**Table S1.** Selected properties of AuNRs with different aspect ratios.

| Sample | Zeta potential  (mV) | UV-Vis absorption(nm)^a)^ | Average AuNRs aspect ratio^b)^ | SERS  intensity^c)^ | S/B ratio^d)^ |
| --- | --- | --- | --- | --- | --- |
| AuNRs-1 | +34.2 | 607 | 1.84 | 3.60 $\times$ 10^4^ | 0.8 |
| AuNRs-2 | +35.8 | 665 | 2.31 | 5.90 $\times$ 10^4^ | 1.1 |
| AuNRs-3 | +37.6 | 743 | 3.26 | 1.04 $\times$ 10^5^ | 1.8 |
| AuNRs-4 | +38.7 | 823 | 4.12 | 1.32 $\times$ 10^5^ | 2.0 |
| AuNRs-5 | +33.5 | 893 | 4.65 | 9.42 $\times$ 10^4^ | 1.6 |

^a)^Absorption peak maximum. ^b)^Determined by transmission electron microscopy. ^c)^Integrated intensity in the 704–764 cm^−1^ range at an adenine concentration of 10^–4^ m. ^d)^Signal-to-background ratio defined as (intensity of the desired signal − intensity of the background signal)/intensity of the background signal.

**Table S2.** Selected properties of AuNRs/NMPs with different weight ratios.

| AuNRs/NMPs (weight ratio)^a)^ | Zeta potential (mV) | SERS intensity^b)^ | S/B ratio^c)^ |
| --- | --- | --- | --- |
| 5/1 | +36.3 | 8.26 $\times$ 10^4^ | 1.4 |
| 2/1 | +35.2 | 1.85 $\times$ 10^5^ | 2.2 |
| 1/1 | +32.5 | 2.24 $\times$ 10^5^ | 2.4 |
| 1/2 | +23.8 | 1.38 $\times$ 10^5^ | 2.0 |
| 1/5 | +14.7 | 4.22 $\times$ 10^4^ | 1.0 |

^a)^HAuCl_4_/clay. ^b)^Integrated intensity in the 704–764 cm^−1^ range at an adenine concentration of 10^–4^ m. ^c)^Signal-to-background ratio defined as (intensity of the desired signal − intensity of the background signal)/intensity of the background signal.

**Table S3.** SERS responses of AuNRs/NMPs/ZnO QDs with different weight ratios to adenine.

| AuNRs/NMPs/ZnO QDs (weight ratio)^a)^ | SERS intensity^b)^ | S/B ratio^c)^ |
| --- | --- | --- |
| 3/3/1 | 1.68 $\times$ 10^5^ | 1.8 |
| 2/2/1 | 1.96 $\times$ 10^5^ | 2.1 |
| 1/1/1 | 2.43 $\times$ 10^5^ | 2.6 |
| 1/1/2 | 2.88 $\times$ 10^5^ | 2.8 |
| 1/1/3 | 2.15 $\times$ 10^5^ | 2.3 |

^a)^HAuCl_4_/clay/ZnO. ^b)^Integrated intensity in the 704–764 cm^−1^ range at an adenine concentration of 10^–4^ m. ^c)^Signal-to-background ratio defined as (intensity of the desired signal − intensity of the background signal)/intensity of the background signal.

**Table S4.** Comparative analysis of SERS-based adenine detection performance among different nanohybrid materials.

| Hybrid composition | SERS intensity^a)^ | LOD^b)^ | EF value^c)^ | S/B ratio^d)^ |
| --- | --- | --- | --- | --- |
| AuNRs | 1.32 $\times$ 10^5^ | 10^-7^ m | 5.8 $\times$ 10^6^ | 2.0 |
| ZnO QDs | 4.43 $\times$ 10^4^ | 10^-5^ m | 7.5 $\times$ 10^4^ | 1.3 |
| AuNRs/NMPs | 2.24 $\times$ 10^5^ | 10^-9^ m | 2.0 $\times$ 10^8^ | 2.4 |
| AuNRs/NMPs/ZnO QDs | 2.88 $\times$ 10^5^ | 10^-10^ m | 1.6 $\times$ 10^9^ | 2.8 |

^a)^Integrated intensity in the 704–764 cm^−1^ range at an adenine concentration of 10^–4^ m. ^b)^Limit of detection. ^c)^SERS enhancement factor calculated as described in the main text. ^d)^Signal-to-background ratio defined as (intensity of the desired signal − intensity of the background signal)/intensity of the background signal.

**Table S5.** Assignments of the peaks in the SERS response to *H. pylori*.

| Raman shift (cm^−1^) | Peak assignments^a)^ | References |
| --- | --- | --- |
| 690 | Guanine ring of DNA/RNA | ^[4]^ |
| 730 | *N*-Acetyl-D-glucosamine | ^[4–6]^ |
| 906 | C–C stretch and skeletal bend | ^[7]^ |
| 1000 | Protein (phenylalanine) | ^[8,9]^ |
| 1330 | CH_2_/CH_3_ wagging of purine bases in nucleic acids | ^[9,10]^ |
| 1461 | C–H deformation of lipids | ^[11]^ |
| 1543 | Tryptophan and phenylalanine; COO^−^, amide II;  N–H bending and C–N stretching | ^[12]^ |
| 1735 | C=O stretch | ^[13]^ |
| 2940 | C–H stretching vibrations of CH_2_ and CH_3_ groups | ^[14,15]^ |
| 3345 | O–H stretch of bound water molecules | ^[16]^ |
| 3511 | O–H stretch of bulk water | ^[17]^ |

^a)^The peak near 690 cm^−1^ was attributed to the C–C twist of guanine rings in DNA/RNA.^[4]^ All SERS responses to *H. pylori* exhibited a peak at 730 cm^−1^ characteristic of *N*-acetyl-D-glucosamine, a component of the bacterial cell wall.^[4–6]^ The peak near 906 cm^−1^ was attributed to C–C stretching and backbone bending.^[7]^ The peak near 1000 cm^−1^ was assigned to phenylpropanoic acid in proteins.^[8,9]^ The peak near 1330 cm^−1^ was attributed to the CH_2_/CH_3_ wobbling of purine in nucleic acids.^[9,10]^ The peak near 1461 cm^−1^ was ascribed to the C–H deformation of lipids.^[11]^ The peak near 1543 cm^−1^ was ascribed to tryptophan and phenylpropanoic acids, COO^–^, amides, and N–H bending and C–N stretching.^[12]^ The peak at 1735 cm^−1^ was attributed to C=O stretching.^[13]^ The broad band at 2940 cm^−1^ (the most intense of all peaks) was ascribed to the stretching vibration of the CH_2_/CH_3_ groups in all macromolecules^.[14,15]^ The O–H stretch of bonded water molecules was observed near 3345 cm^−1^,^[16]^ while that of bulk water was observed near 3511 cm^−1^.

**Table S6.** Performances of different gold nanoparticle–based probes for the SERS detection of adenine and bacteria.

| Hybrid composition^a)^ | Analyte molecule | SERS  LOD^b)^/EF value^c)^ | References |
| --- | --- | --- | --- |
| AuNPs | Adenine | 3.5 $\times$ 10^-8^ m/1.0 $\times$ 10^6^ | ^[18]^ |
| AuNPs/Silicate |  | 10^-9^ m/Not given | ^[19]^ |
| AuNPs/MoWS_2_ |  | 10^-9^ m/Not given | ^[20]^ |
| AuNRs/Ti_3_C_2_T_x_ |  | 10^-9^ m/Not given | ^[21]^ |
| TAuNPs/NMPs |  | 10^-9^ m/5.7 $\times$ 10^7^ | ^[22]^ |
| AuNCs/NMPs |  | 10^-9^ m/3.6 $\times$ 10^8^ | ^[2]^ |
| AuNRs/NMPs/ZnO QDs |  | 10^-10^ m/1.6 $\times$ 10^9^ | Our work |
| M13-AuNPs^d)^ | *S. aureus* | 10 CFU mL^−1^ | ^[23]^ |
| AuNPs/PDMS^e)^ |  | 13 CFU mL^−1^ | ^[24]^ |
| Gold nanoflowers |  | 10^3^ CFU mL^−1^ | ^[25]^ |
| TAuNPs/NMPs |  | 10^2^ CFU mL^−1^ | ^[22]^ |
| AuNCs/POE/NMPs |  | 92 CFU mL^−1^ | ^[2]^ |
| AuNRs/NMPs/ZnO QDs |  | 10 CFU mL^−1^ | Our work |
| Au@AgNRs | *E. coli* | 10^2^ CFU mL^−1^ | ^[26]^ |
| AuNCs/PIB–POE–PIB/NMPs |  | 1.6 $\times$ 10^2^ CFU mL^−1^ | ^[2]^ |
| AuNRs/NMPs/ZnO QDs |  | 50 CFU mL^−1^ | Our work |

^a)^AuNPs = gold nanoparticles; TAuNPs = triangular gold nanoplates; AuNCs = gold nanocubes; Au@AgNRs = gold@silver core-shell nanorods. ^b)^Limit of detection. ^c)^SERS enhancement factor calculated as described in the main text. ^d)^M13 phage with specific *S. aureus*-binding heptapeptide displayed on the N-terminal of pIII protein is selected from the phage display peptide library. ^e)^An aptamer (Apt 1) was modified on the substrate through the Au–S bond as the capture probes.

**References**

1. J. Zheng, X. Cheng, H. Zhang, X. Bai, R. Ai, L. Shao, J. Wang, *Chem. Rev.* **2021**, *121*, 13342.
2. Y.-F. Chen, M.-C. Lu, C.-J. Lee, C.-W. Chiu, *J. Mater. Chem. B* **2024**, *12*, 3226.
3. M. Izzi, M. C. Sportelli, L. Torsi, R. A. Picca, N. Cioffi, *ACS Appl. Nano Mater.* **2023**, *6*, 10881.
4. J. Sundaram, B. Park, Y. Kwon, K. C. Lawrence, *Int. J Food Microbiol.* **2013**, *167*, 67.
5. R. M. Jarvis, R. Goodacre, *Anal. Chem.* **2004**, *76*, 40.
6. S. Liu, Y. Zhu, L. Zhao, M. Li, D. Liang, M. Li, G. Zhao, Y. Ma, Q. Tu, *Anal. Chim. Acta* **2024**, *1308*, 342616.
7. R. Ahijado-Guzmán, P. Gómez-Puertas, R. A. Alvarez-Puebla, G. Rivas, L. M. Liz-Marzán, *ACS Nano* **2012**, *6*, 7514.
8. D. Franco, L. De Plano, M. Rizzo, S. Scibilia, G. Lentini, E. Fazio, F. Neri, S. Guglielmino, A. Mezzasalma, *Spectrochim. Acta A Mol. Biomol. Spectrosc.* **2020**, *224*, 117394.
9. E. Witkowska, K. Niciński, D. Korsak, T. Szymborski, A. Kamińska, *Anal. Bioanal. Chem.* **2019**, *411*, 2001.
10. D. P. Lau, Z. Huang, H. Lui, D. W. Anderson, K. Berean, M. D. Morrison, L. Shen, H. Zeng, *Lasers Surg. Med.* **2005**, *37*, 192.
11. M. Kashif, M. I. Majeed, H. Nawaz, N. Rashid, M. Abubakar, S. Ahmad, S. Ali, H. Hyat, S. Bashir, F. Batool, S. Akbar, M. A. Anwar, *Spectrochim. Acta A Mol. Biomol. Spectrosc.* **2021**, *261*, 119989.
12. Prakash, S. Sil, T. Verma, S. Umapathy, *J. Phys. Chem. C* **2019**, *124*, 861.
13. K. Tanaka, R. Oketani, T. Terada, P. Leproux, Y. Morono, H. Kano, *J. Phys. Chem. B* **2023**, *127*, 1940.
14. D. Kusić, B. Kampe, A. Ramoji, U. Neugebauer, P. Rösch, J. Popp, *Anal. Bioanal. Chem.* **2015**, *407*, 6803.
15. Y. S. Mary, K. Raju, I. Yildiz, O. Temiz-Arpaci, H. I. Nogueira, C. M. Granadeiro, C. Van Alsenoy, *Spectrochim. Acta A Mol. Biomol. Spectrosc.* **2012**, *96*, 617.
16. R. Vyumvuhore, A. Tfayli, H. Duplan, A. Delalleau, M. Manfait, A. Baillet-Guffroy, *Analyst* **2013**, *138*, 4103.
17. M. E. Sohová, M. Bodík, P. Siffalovic, N. Bugárová, M. Labudová, M. Zaťovičová, T. Hianik, M. Omastová, E. Majková, M. Jergel, S. Pastoreková, *Analyst* **2018**, *143*, 3686.
18. J. Zhou, D. Wang, H. Yang, F. Wang, *Spectrochim. Acta A Mol. Biomol. Spectrosc.* **2022**, *270*, 120801.
19. Y.-C. Lee, C.-W. Chiu, *Nanomaterials* **2019**, *9*, 324.
20. X. Zhang, Y. Li, Z. Lei, Y. Yang, L. Tao, Z. Zheng, X. Feng, J. Zeng, Y. Zhao, *ACS Appl. Nano Mater.* **2023**, *6*, 16000.
21. P. F. Wu, X. Y. Fan, H. Y. Xi, N. Pan, Z. qian Shi, T. T. You, Y. K. Gao, P. G. Yin, *J. Alloys Compd.* **2022**, *920*, 165978.
22. Y.-F. Chen, W.-R. Chang, C.-J. Lee, C.-W. Chiu, *J. Mater. Chem. B* **2022**, *10*, 9974.
23. X.-Y. Wang, J.-Y. Yang, Y.-T. Wang, H.-C. Zhang, M.-L. Chen, T. Yang, J.-H. Wang, *Talanta* **2021**, *221*, 121668.
24. A. Zhu, S. Ali, Y. Xu, Q. Ouyang, Q. Chen, *Biosens. Bioelectron.* **2021**, *172*, 112806.
25. S. Juneja, J. Bhattacharya, *Colloids Surf. A* **2019**, *182*, 110349.
26. L. Bi, X. Wang, X. Cao, L. Liu, C. Bai, Q. Zheng, J. Choo, L. Chen, *Talanta* **2020**, *220*, 121397.
